# Identification and characterization of two salmon louse heme peroxidases and their potential as vaccine antigens

**DOI:** 10.1016/j.isci.2023.107991

**Published:** 2023-09-21

**Authors:** Elisabeth Gislefoss, Amr Ahmed Abdelrahim Gamil, Aina-Cathrine Øvergård, Øystein Evensen

**Affiliations:** 1 Faculty of Veterinary Medicine, Norwegian University of Life Sciences, Ås, Norway; 2 Department of Biological Sciences, University of Bergen, Bergen, Norway; 3 Sea Lice Research Center, University of Bergen, Bergen, Norway

**Keywords:** Biological sciences, Immunology, Parasitology, Phylogeny

## Abstract

Salmon louse, *Lepeophtheirus salmonis,* represents major challenge for salmon farming. Current treatments impose welfare issues and are costly, whereas prophylactic measures are unavailable. Two salmon louse heme peroxidases (LsPxtl-1 and LsPxtl-2) were tested for their importance for parasite development and as potential vaccine candidates. LsPxtl-1 possesses two heme peroxidase domains and is expressed in ovaries and gut, whereas LsPxtl-2 encodes one domain and contains N-terminal signal peptide and an Eph receptor ligand-binding domain. LsPxtl-1, but not LsPxtl-2, knockdown in nauplius II stage caused poor swimming and death, indicating its importance for parasite development. Immunizations using single DNA plasmid injection encoding the peroxidases or heterologous prime (DNA) and boost (recombinant LsPxtl-2 protein) gave non-significant reduction in lice numbers. Single injection gave low specific antibody levels compared with the prime-boost. The findings suggest LsPxtl-1 is important for parasite development but formulations and vaccination modalities used did not significantly reduce lice infestation.

## Highlights

• LsPxtl-1 is important for normal development of salmon louse
• DNA immunization with LsPxtl-1 and -2 raises specific antibodies in salmon
• Recombinant protein boost immunization improves the antibody response
• Prime-boost-vaccinated fish had non-significantly reduced lice counts postchallenge

## Graphical abstract

**Figure:**
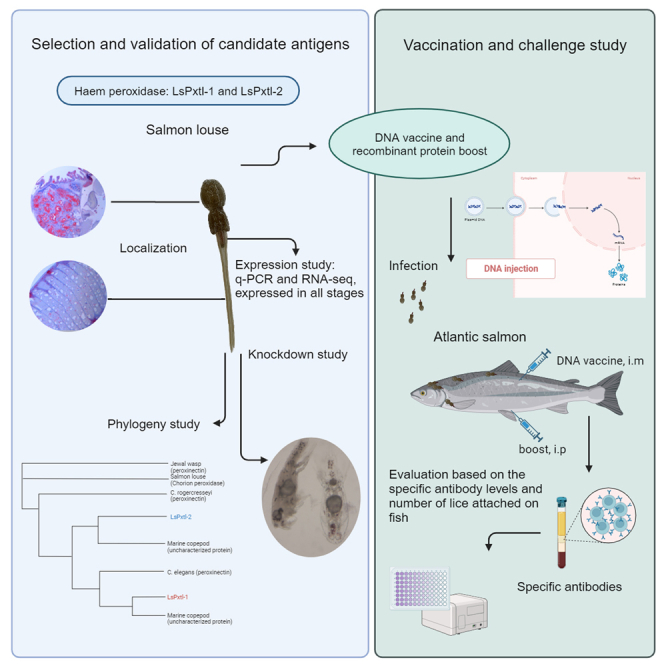

## Introduction

The ectoparasitic salmon louse (*Lepeophtheirus salmonis*) is part of the marine fauna in the Northern hemisphere. It belongs to the phylum Arthropoda, class Hexanauplia, and family Caligidae. Atlantic salmon (*Salmo salar* L.) and brown trout (*Salmo trutta*) are important hosts, whereas the pacific salmonids Coho (*Oncorhynchus kisutch*) and pink salmon (*Oncorhynchus gorbuscha*) are also subjected to infestation but are highly resistant compared with Atlantic salmon. The life cycle consists of 8 developmental stages, starting with two free-living nauplius stages after which the parasite molts into copepodid, the first parasitic stage. The copepodids dock onto the skin, fins, and gills with their second antenna before attaching by a frontal filament just before molting into the chalimus stages. It continues to stay attached by this filament through two chalimus stages, until the two preadult stages, and finally the adult stage where it can produce new offspring.^1^^,^^2^^,^^3^ The louse feeds on skin, mucus, and blood, causing wounds, stress, and reduced health, and can thereby facilitate secondary infestation with other pathogens.^4^^,^^5^^,^^6^^,^^7^

Salmon louse imposes huge costs to the Norwegian salmon farming industry; an estimated cost ranges between 8 and 14 NOK/kg produced, equivalent to around 10.8–19 billion NOK (1.1–1.9 billion USD)^8^ mainly due to the cost of treatment. Several treatment strategies such as biological control, functional feed, as well as chemical, thermal, mechanical, and freshwater treatment have been used to keep the lice infestation levels below 0.5 adult females/fish in average per cage and below 0.2 adult females/fish during periods of wild salmon smolt migration. Chemical treatment was the method of choice up to 2015 but resistance to most of the chemicals has rendered it an ineffective treatment.^9^^,^^10^^,^^11^^,^^12^^,^^13^^,^^14^^,^^15^ In the recent years, various non-medicinal treatments have been developed as an alternative but has resulted in new problems with increased posttreatment mortalities and ethical concerns related to use of cleaner fish.^16^ As a result, the lice problem has exacerbated, and there is need for new control strategies, preferably prophylactic approaches, such as vaccination.

Developing vaccines against an ectoparasite is challenging. In general, ectoparasites are less exposed to the immune system, and efforts to control infestation through immunization would rely on immune effectors having an impact on attachment, development, and/or maturation of the parasite while it is feeding on the host. Defining the “correct” antigen is a needle-in-the-haystack exercise, and which concept to use is another challenge. A concealed antigen strategy has been reasonably successful for blood-sucking parasite in cattle,^17^^,^^18^ where the concealed antigen is hidden from the host immune systems and made accessible through vaccination. Vaccines using this strategy usually elicit adaptive immune responses and exert its effect when the parasite feeds blood. There are currently commercially available vaccines against *Boophilus microplus* (cattle tick) that have been developed using this strategy.^19^^,^^20^ However, the current vaccines have shown unequal or low effect on several tick species, such as the Ixodes tick. New vaccine models that can work against several tick species to obtain effective control have been studied by looking into tick biology, reverse genetics, tick protein evolution, and vaccinomics. Different studies in the tick have been focused on antigens associated with egg, salivary gland, midgut, malpighian tubules, and tick cement. However, there are several factors that play a role on the efficacy. Folding of the protein has shown to be important for several antigens. Some antigens have shown poor efficacy alone, whereas combining them with other antigens improves protection. Candidate antigens consisting of conserved proteins are suggested to have the ability to produce cross-reaction to several tick species. Heterologous vaccination may also improve protection. It is thought that a DNA vaccine may give good cross-protection in the host against several ticks if boosted with a recombinant protein or chimeric vaccine.^21^^,^^22^ Because the salmon louse share many of the features with the mentioned parasites, a similar strategy, using concealed antigen, can potentially be used to develop vaccines against salmon louse.

Earlier studies have documented that Atlantic salmon mount an antibody response after a natural lice infestation,^23^ which has prompted several attempts to develop vaccines against the salmon louse with low or moderate success. Ross et al. developed a recombinant vaccine targeting a salmon louse trypsin and reported a reduction in the number of attached lice starting at day 14 postinfestation (p.i.).^24^ Another recombinant vaccine targeting the vitellogenin in the parasite resulted in lower number of attached female lice and less erosion or wounds on the vaccinated fish compared with the unvaccinated control.^25^ Furthermore, vaccines developed using a recombinant peptide from the ribosomal protein P0 resulted in reduction in attached female lice and their ability to produce eggstrings that supposedly continued into the F1 lice generation.^26^ A recent study showed that a recombinant lice gut protein named P33 elicited specific antibodies after immunization and resulted in reduction of immature and adult stages of salmon lice.^27^ Casuso et al. tested recombinantly express proteins from *Caligus rogercresseyi*, peritrophin, cathepsin, or a mix of the two, in a vaccine trial and obtained a reduction in infestation of 24%, 44%, and 52%, respectively, compared with non-vaccinated controls.^28^ All these studies have provided some degree of documentation of proof of a principle, but the protection obtained has been relatively low or moderate, and testing at large scale has not been reported.

In search for vaccine candidates against lice, it is assumed that two criteria must be fulfilled for a successful outcome: (1) the antigens should be crucial for lice survival, development, or interaction with the host, and (2) they have to be delivered in a form that will elicit protective host immune responses. We have employed a rational approach where the first criterion was addressed by testing the importance of candidate molecules for lice survival or development through gene knockdown studies in early developmental stages of lice. An optimized immunization protocol included injection of plasmid encoding target genes, combined with a heterologous boost using the expressed protein based on previous studies showing that heterologous prime-boost vaccination^29^ broadens the immune response.

Peroxidases are candidate proteins that possibly fulfill the above-mentioned criteria because of their importance for the parasite’s immune responses, defense mechanism, and pathogenicity. Heme peroxidases are a group of peroxidases consisting of three closely related subfamilies, namely peroxidasins, peroxinectins, and chordata peroxidases, that are potential vaccine candidates due to their variant and important functions.^30^ For example, *Drosophila* peroxidasins were found to be secreted both in larvae and adults and to play important and diverse roles in the extracellular matrix formation, phagocytosis, defense mechanism, and development.^31^^,^^32^ A Drosophila peroxinectin-like gene, curly suppressor, was identified as an important factor necessary for wing maturation,^33^ and a crayfish peroxinectin was found to function as a cell adhesion ligand.^34^ A dual oxidase in Drosophila has been shown to have a role in gut immunity, as knock down of the protein caused an increase of mortality when exposed to infestation, illustrating that the heme peroxidase also functions on the mucosal epithelial barrier as well as being implicated in phagocytosis.^35^ In *Anopheles gambiae*, a peroxidase/dual oxidase system is involved in the insect immune responses, especially modulation of the midgut epithelium. This peroxidase/dual oxidase system decreases gut permeability to immune effectors, giving optimal environment for the microbiota and the malaria parasite.^36^ Perhaps the most interesting/compelling features that make peroxidase interesting vaccine antigens against blood sucking parasites are their involvement in combating heme toxicity and in important redox metabolism pathways that can affect the parasite infectivity and survival.^37^ Studies have shown that the peroxiredoxin Salp25D in the *Ixodes scapularis* may detoxify ROS at the feeding site and in the tick gut, whereas the heme peroxidase 2/NADPH oxidase 5 system has an important role in epithelium nitration in the mosquito. In the *Rhipicephalus Microplus*, the glutathione peroxidase is part of the antioxidant system that help regulate the expression of proteins involved in ROS detoxification.^38^

Heme peroxidases have also been identified in the salmon louse, and there are around 25 potential heme peroxidase genes predicted within its genome.^39^ There is, however, still a knowledge gap related to the function of these peroxidases, as very few have been characterized in terms of function. One gene that has been investigated, named *Lepeophtheirus salmonis* heme peroxidase 1 (*LsHPX1*), showed similarities to chorion peroxidase and genes taking part in cuticle hardening and adhesion.^40^ Poor swimming ability was seen in copepodids after knock down (KD) of the gene in nauplius, and based on histological changes of KD animals, a role in crosslinking molecules of the extracellular matrix was suggested. Another heme peroxidase gene, LsPxt1, was found to be expressed by an exocrine gland type believed to be important for integument lubrication, but its function has not been elucidated.^41^ In the current study, we have identified and characterized two additional salmon louse peroxidase genes and investigated their importance for lice survival by performing RNAi experiments. The ability of the two peroxidases to elicit protective immune responses in Atlantic salmon was tested in subsequent vaccination and challenge trial.

## Results

### Identification, characterization, and phylogenetic analysis

**Figure 1:**
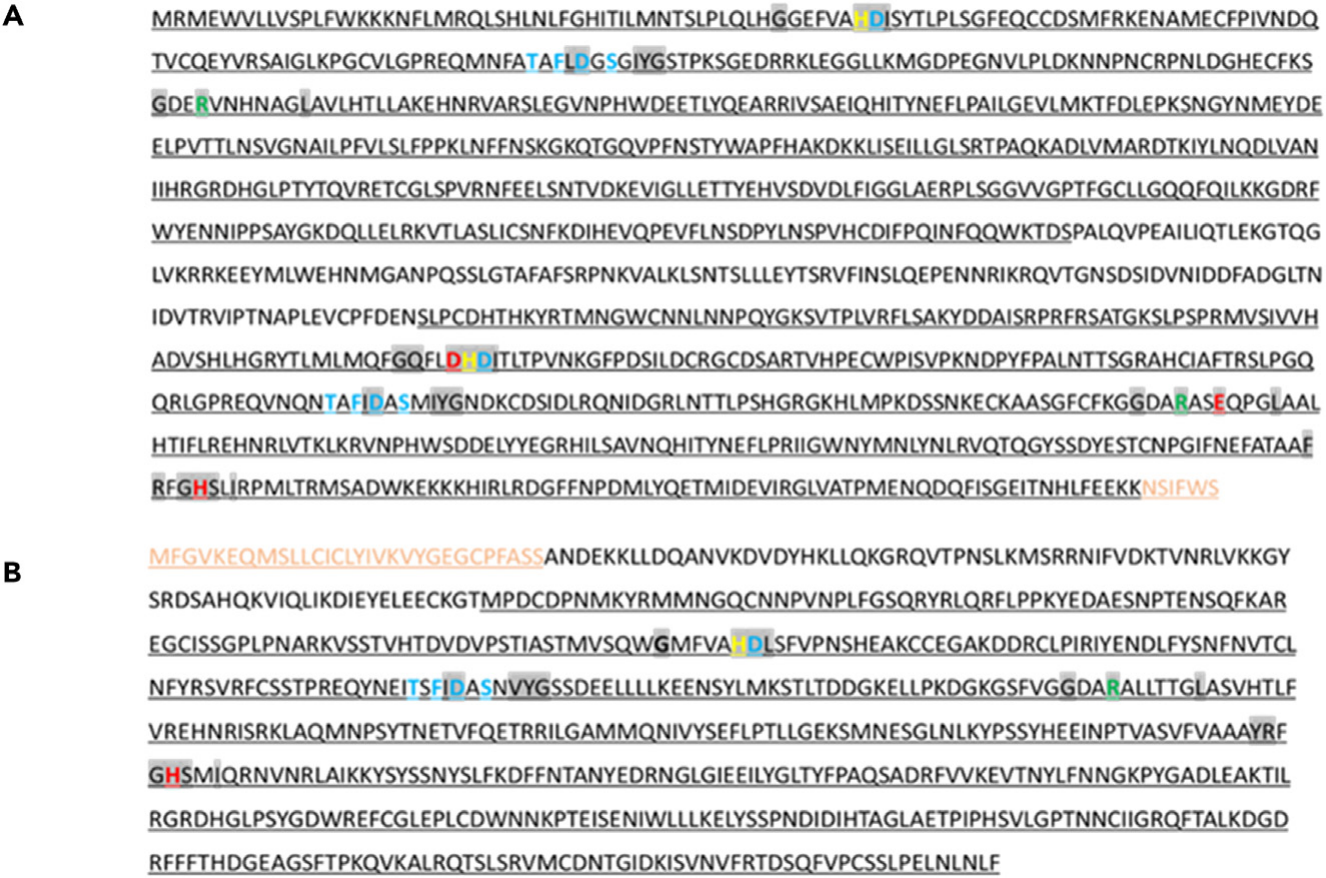
Graphic presentation of the identified residues in the LsPxtl-1 and 2 (A and B) The identified peroxidase domains are underlined, two for LsPxtl-1 (A) and one for LsPxtl-2 (B). Conserved residues typical for peroxidase-cyclooxygenase superfamily are highlighted with gray. Red indicates heme-binding sites, whereas blue indicates Ca^2+^-binding sites. Yellow indicates proton acceptor site, and green shows a transitions state stabilizer. Light brown sequence indicates a predicted LDLRB repeat profile in A and Eph receptor ligand-binding domain profile in B. See also Figure S1 and S2.

A partial DNA sequence of 3,186 nucleotides without a poly A tail was obtained for LsPxtl-1 (LsPxtl-1 OP649849), though covering an open reading frame of 1,060 amino acids (Figure S1). Comparing the sequence with genes found in other species by blast searches in the UniProt database,^42^ LsPxtl-1 was found to be similar to sequences annotated as uncharacterized or peroxidasin in species within the phylum Arthropoda, mainly subphylum Hexapoda and Crustacea (Table S1). A blast search in the Redoxibase,^43^ a specialized peroxidase database, revealed similarities with heme peroxidases within species belonging to phylum Nematoda, Arthropoda, and Echinodermata. Most closely related enzymes are identified as peroxinectin but also a few as peroxidasin (Table S2). The motif analysis showed that the protein has two peroxidase domains, belonging to the animal heme peroxidase superfamily. Five Ca^2+^-binding sites characteristic of family 3 of the peroxidase-cyclooxygenase superfamily were identified in both domains (Figure 1A). This superfamily contains mainly peroxinectins, and the protein was accordingly named *Lepeophtheirus salmonis* Peroxinectin-like 1 (LsPxtl-1). No heme-binding sites were identified within the first peroxidase domain, whereas the second domain contained three residues known to be involved in heme binding in the Prosite database. On the contrary, the InterPro database identified several heme-binding sites in the first domain and none in the latter.^44^^,^^45^ The classical consensus distal sequences X-G-Q-X-X-D-H-D-X and X-R-X-X-E-X were only present in the second domain that also contained a proximal X-G-H-S-X-I sequence (Figure 1A). In contrast, the first domain contained the following patterns: X-G-G-X-X-A-H-D-X and X-R-X-X-H-X. In addition to the peroxidase domains, a low-density lipoprotein receptor class B (LDLRB) repeat profile was identified at the C terminus but with low confidence in the Prosite database.

A full DNA sequence for LsPxtl-2 (LsPxtl-2, OP649850) was obtained, with RACE consisting of 1,986 nucleotides (Figure S2). A relatively shorter open reading frame than that of LsPxtl-1 was identified in LsPxtl-2, encoding 661 amino acids. Blasting the sequence in UniProt^42^ revealed that the top 25 species with highest identity belonged to phylum Arthropoda and most of them to subphylum Hexapoda with nucleotide sequence coding for peroxidasin, chorion peroxidase, and peroxidase-like proteins (Table S3). Comparing the protein sequence of LsPxtl-2 with other species in the Redoxibase database showed relations to the invertebrate peroxinectin belonging to the phylum Arthropoda, class Insecta and Malacostraca, and eosinophil peroxidase belonging to phylum Chordata, class Mammalia (Table S4). Unlike LsPxtl-1, the motif analysis identified only a single peroxidase domain (Figure 1B), belonging to the animal heme peroxidase superfamily. The protein was also predicted to contain a signal peptide on the N terminal of the protein in the InterPro database, whereas an Ephrin (Eph) receptor ligand-binding domain profile was detected in the same sequence stretch using the Prosite database but with weak confidence. Similar to LsPxtl-1, the heme-binding domain contained five Ca^2+^-binding sites and was designated as *Lepeophtheirus salmonis* Peroxinectin-like 2 (LsPxtl-2). The heme-binding site of LsPxtl-2 also lacked the classical X-G-Q-X-X-D-H-D-X and X-R-X-X-E-X distal sequences but contained a proximal X-G-H-S-X-I sequence (Figure 1B). The patterns X-G-M-X-X-A-H-D-X and X-R-X-X-L-X were observed for the distal sequences.

**Figure 2:**
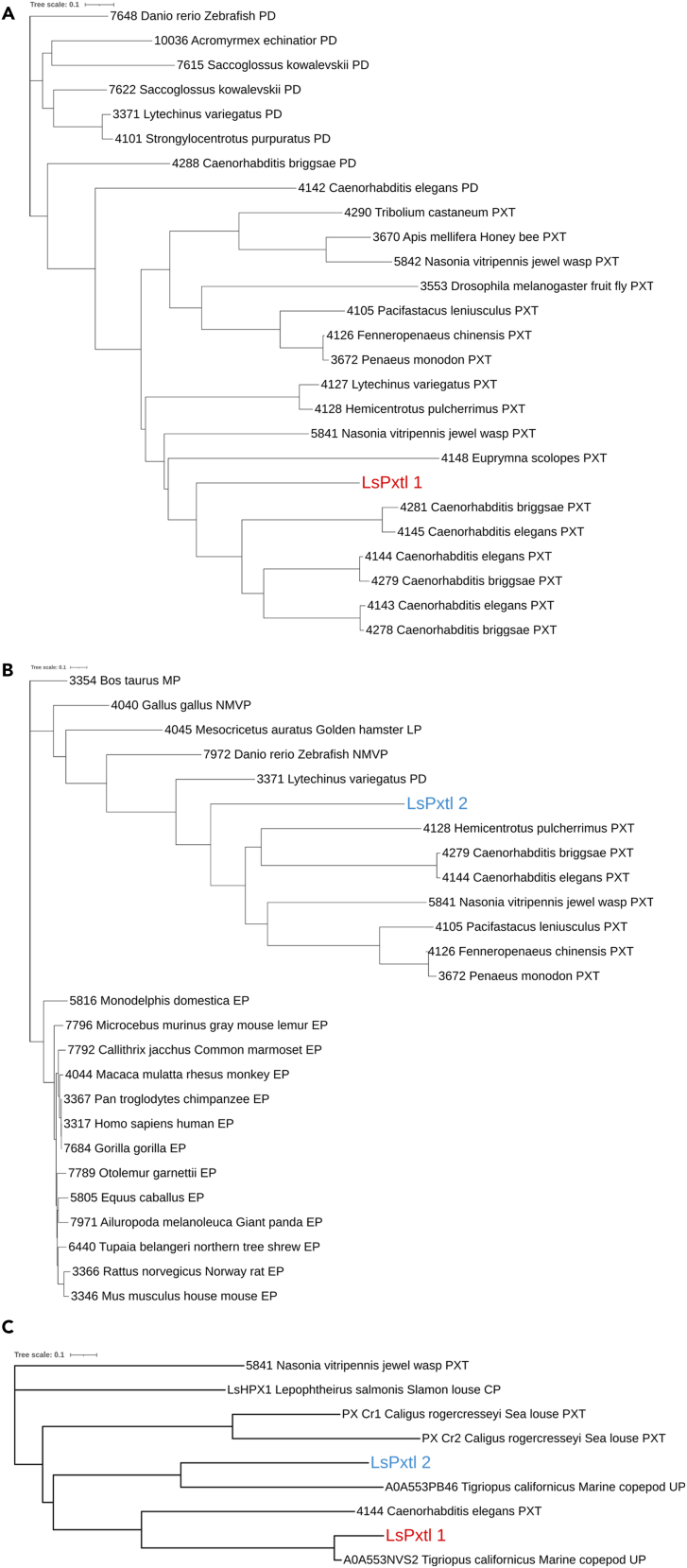
Phylogenetic tree illustrating the relations between the identified proteins and proteins of different species Protein sequences of both variants were blasted in the UniProt (Figure S3; Tables S1 and S3) and Redoxibase database (Tables S2 and S4), and top 25 proteins were added to the tree based on highest identity and E-value. (A‒C) (A) LsPxtl-1 Redoxibase, (B) LsPxtl-2 redoxibase, and (C) mix between LsPxtl-1 and 2 and top result from UniProt and Redoxibase and a characterized peroxidase in the salmon louse, LsHPX1, and two peroxinectins, PX-Cr1 and PX-Cr2, in sea louse *Caligus rogercresseyi*. PXT, peroxinectin; PD, peroxidasin; MP, myeloperoxidase; NMVP, non-mammalian vertebrate peroxidase; LP, lactoperoxidase; EP, eosinophil peroxidase; CP, chorion peroxidase; UP, uncharacterized protein. The illustration tree is made by NGPhylogeny.fr.^46^ See also Tables S2 and S4.

Next, a phylogenetic analysis, performed for both genes in NGPhylogeny.fr using data obtained in UniProt (Figure S3) and Redoxibase (Figure 2), was conducted to study the relationship between the LsPxtl genes and selected heme peroxidases of other species. The analysis performed with sequences obtained by BLAST searches in the UniProt database showed that LsPxtl-1 seems to have the highest resemblance to an uncharacterized protein in the marine copepod *Tigriopus californicus*, with an identity of 72.2% (Table S1). Two uncharacterized proteins in *Daphnia magna* and *Daphnia pulex*, species of water fleas belonging to the subphylum Crustacea and class Branchiopoda, are also found to be closely related to the LsPxtl-1 but with lower identity scores of 49.2% and 48.4%, respectively. A motif analysis (Prosite) of the protein sequences for these uncharacterized proteins revealed that they also encode two peroxidase domains, similar to LsPxtl-1. Most of the other identified proteins belonged mainly to species within the Hexapoda subphylum. However, two peroxidasins with around 46% identity to LsPxtl-1 were found in the springtails: *Orchesella cincta* and *Folsomia candida* belonging to class Collembola. Another peroxidasin and a chorion peroxidase were found in species of class Insecta, with an identity score of 44%–45% (Table S1). In contrast, the phylogenetic analysis conducted based on sequences obtained in Redoxibase indicated that LsPxtl-1 has the highest resemblance to a *Caenorhabditis elegans* peroxinectin protein, with an alignment length of 1,043 and an identity score of 35%. Most of the other identified proteins were peroxinectins identified in shrimps, wasp, bee, and fruit fly. In addition, peroxinectin and peroxidasin belonging to different species of sea urchin were also identified (Table S2).

The phylogenetic tree (Figure S3B) for the UniProt BLAST results identified an uncharacterized protein from *Tigriopus californicus* to have the highest similarity (42% identity) to LsPxtl-2. Springtail peroxidasin and chorion peroxidase were also found to be closely related to LsPxtl-2 with identity scores around 37%–39% (Table S3). The phylogenetic tree constructed with the data obtained from Redoxibase showed, however, that a peroxinectin belonging to the jewel wasp (*Nasonia vitripennis*) within phylum Arthropoda and class Insecta had the highest e-value with an alignment length of 597 and identity score of 34%. Peroxinectins identified in shrimp and one other crayfish belonging to subphylum Crustacea have also been identified. Interestingly, most of the other identified proteins were mammalian eosinophil peroxidases (Table S4). LsPxtl-1 and 2 align with two peroxinectins, PX-Cr1 and PX-Cr2, for *C. rogercresseyi* (Figure 2C).

### Localization of LsPxtl-1 and LsPxtl-2 transcripts

**Figure 3:**
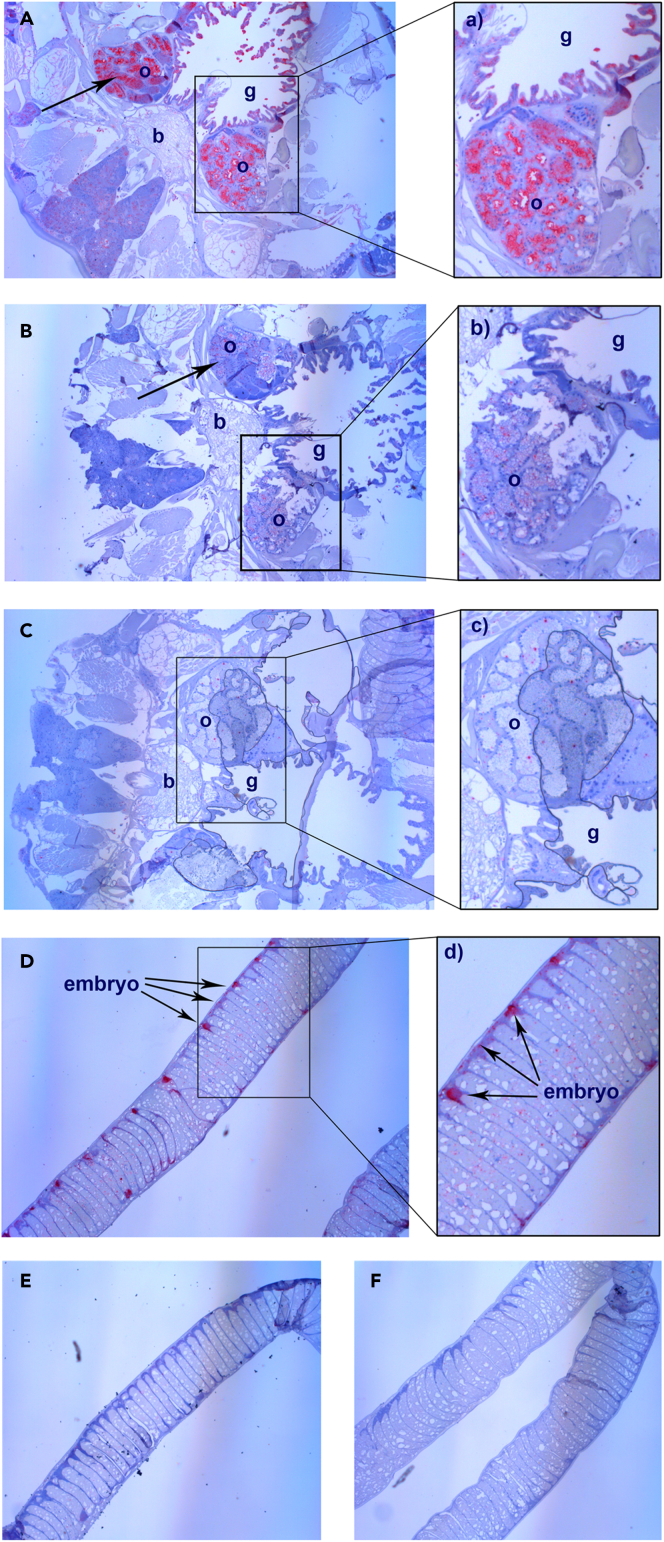
*In situ* hybridization of adult female lice with eggstrings Red coloration illustrates mRNA expression in the tissue. (A‒F) (A) LsPxtl-1 lice body; a: detail of LsPxtl-1 expression. (B) LsPxtl-2 lice body; b: detail of LsPxtl-2 expression. (C) Negative control lice body; c: detail of negative control. (D) LsPxtl-1 eggstring; d: detail showing LsPxtl-1 expression in eggstring. (E) LsPxtl-2 eggstring. (F) Negative control eggstrings. o, ovaries; b, brain; g, gut. Arrows point at coloration.

To investigate the tissue distribution of both genes, RNA probes were applied to sections of an adult female with eggstrings using *in situ* hybridization. Staining for both genes were mainly seen in the ovaries (Figures 3A and 3B), but the expression of LsPxtl-1 (Figure 3Aa) was much higher compared with LsPxtl-2 (Figure 3B), with control staining in Figure 3C. LsPxtl-1 was also found to be expressed in the gut, tegumental type 1 glands and in the developing embryos of the eggstrings (Figure 3D). The expression pattern in the eggstring was localized in areas toward the periphery of the segments (Figure 3D). A similar expression pattern was not seen for LsPxtl-2 (Figure 3E). Negative control is shown in Figure 3F.

### Ontogenetic analysis

**Figure 4:**
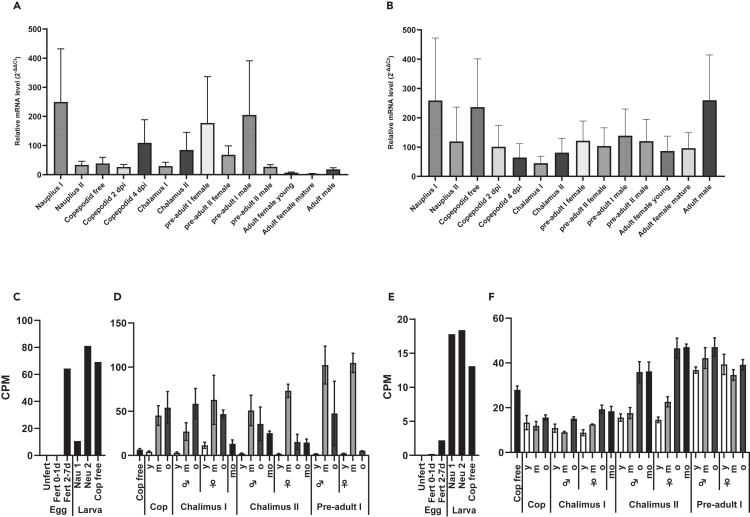
Relative transcript level of LsPxtl-1 and LsPxtl-2 in different salmon louse stages (A–F) Relative transcript level of LsPxtl-1 (A, C, and D) and LsPxtl-2 (B, E, and F) in different salmon louse developmental stages. (A and B) Transcript levels measured by qPCR in all lice life stages; fertilized egg (egg), nauplius (Nau) 1 and 2, planktonic copepodites (cop f), copepodites 2 days (cop 2d) and 4 days (cop 4d) post-infestation of fish, chalimus (chal) 1 and 2, preadult (pad) 1 and 2, adult (ad) young (Y) and mature (M). Expression is given as average 2^−ΔΔCt^ ± SD (N = 5). (C‒F) Detailed ontogenetic expression profile measured by RNA sequencing, presented as average counts per million (CPM) reads. (C and E) Expression in eggs, both unfertilized (unfert) and fertilized (fert) eggs around 1 day and between 2 and 7 days postfertilization, and in nauplius (nau) and planktonic copepodid (cop free) larva (N = 1). (D and F) Expression in planktonic copepodids (cop free), but also in young (y), middle (m), old (o) and for some stages molting (mo) copepodids (cop), chalimus 1, chalimus 2, and preadult 1, given as CPM ±SD (N = 3).

The relative transcript level of LsPxtl-1 and LsPxtl-2 analyzed by real-time PCR in different louse developmental stages showed constitutive expression, with variation, for both variants throughout all developmental stages. The expression of LsPxtl-1 in nauplius I is highest and declines in the following stage until copepodite, 4 days postinfestation (dpi, Figure 4A), with a similar trend for LsPxtl-2, with expression being highest in early developmental stages (Figure 4B). In contrast to LsPxtl-1, adult male also had a high expression level of LsPxtl-2.

Analysis of RNA sequencing data of eggstrings and later stages sampled at different time points after fertilization showed increased expression of LsPxtl-1 in older embryos, not seen for LsPxtl-2 (Figures 4C and 4E). Expression of both genes showed high variation, likely linked to variation in rate of development on the host, and each sampling will contain individuals of different developmental stages that might explain the variation observed. Analysis of RNA sequencing data of individual copepodids, chalimus, pre-adult, and adult ages showed a cyclic expression pattern over the molting cycles, with relatively higher expression levels seen in middle and old instar ages (Figures 4D and 4F), most pronounced for LsPxtl-1 (Figure 4D).

### Functional knockdown study

**Figure 5:**
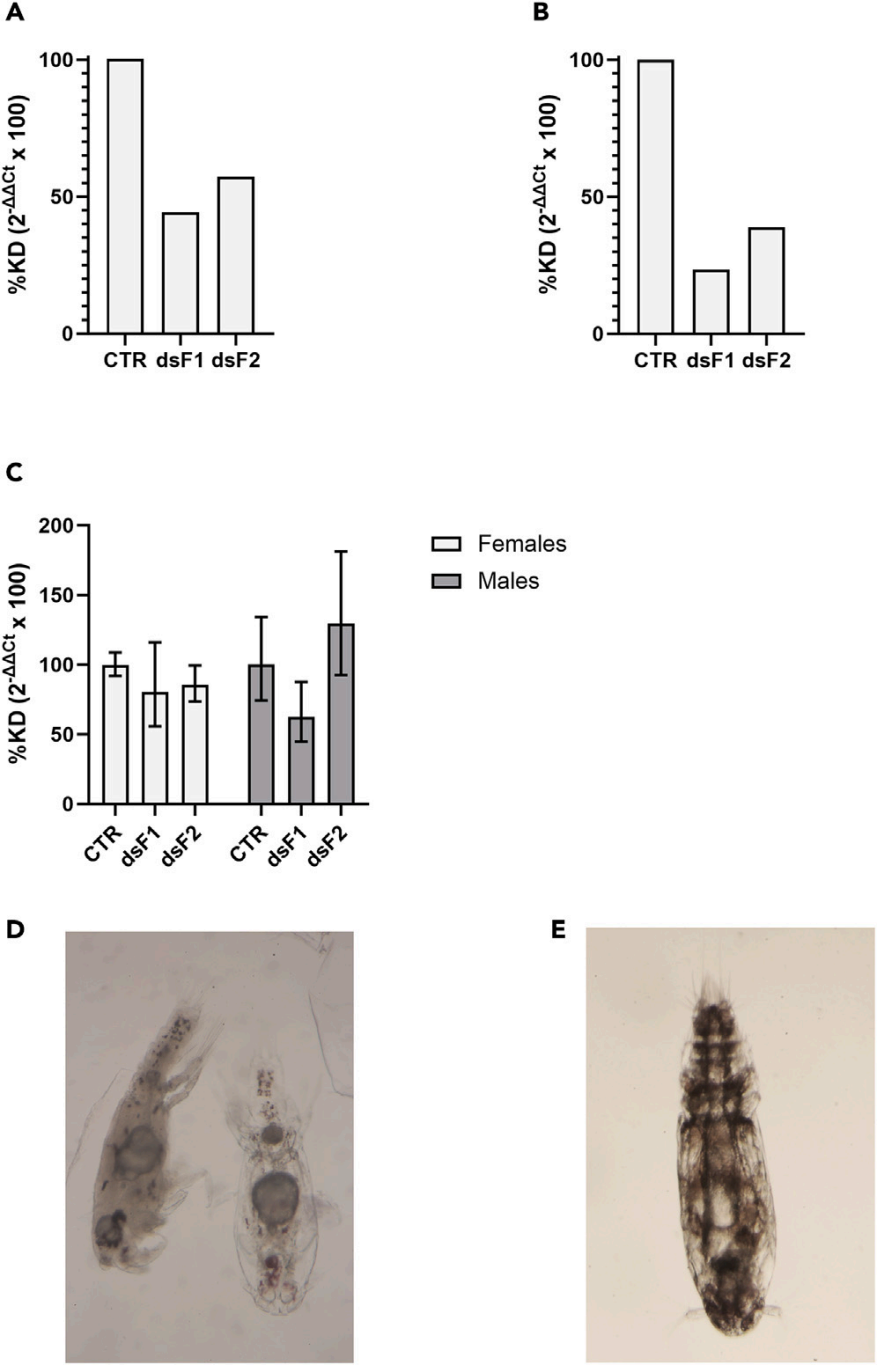
Expression of LsPxtl-1 and LsPxtl-2 in copepodites and preadult after knockdown of genes in nauplius stage Lice were treated with dsRNA fragments; each group received one fragment. (A‒C) Control received a dsRNA CPY control gene The graphs show percent knockdown for the controls and for the copepodites that received two different fragments of (A) LsPxtl-1, (B) LsPxtl-2, and in preadults (C) LsPxtl-2. The figures show downregulation in the copepodite stage, whereas the effect of the knockdown is almost gone at the pre-adult stage for LsPxtl-2, mean with SD. (D) Copepodites with abnormalities seen as dense, circular zone in the intestinal region after knockdown of LsPxtl-1. (E) Control copepodites.

**Figure 6:**
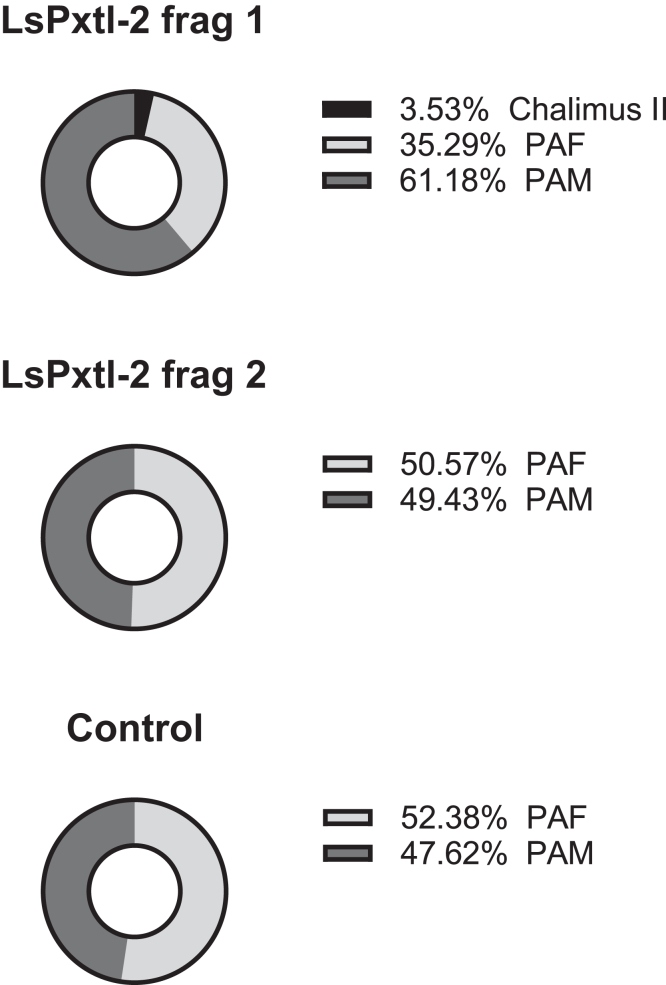
RNAi knockdown of nauplii for LsPxtl-2 followed by infestation of knocked down lice on Atlantic salmon The figures show the percentage of the different life stages and gender collected on the fish after termination of RNAi experiment (LsPxtl-2). PAF, pre-adult female; PAM, pre-adult male; frag, fragment.

An RNAi study was then undertaken to understand the functional importance of the identified genes. Knockdown of LsPxtl-1 in nauplius II resulted in death shortly after molting into copepodites (Figure 5A). The copepodites showed an abnormal growth in the gut region (Figure 5D), lack of coordination, and lost their ability to swim shortly before dying. In contrast, no phenotypic changes were observed when RNAi was performed on pre-adult II lice for LsPxtl-1. Knockdown of LsPxtl-2 gave no phenotypic changes in nauplius II or copepodids (Figures 5B and 5C; Table 1; Figure 6) and also not in injected pre-adult II lice. Control lice treated with double-stranded RNA (dsRNA) CPY control gene (non-target species) behaved normally, and no phenotypic changes were observed (Figure 5E).

**Table 1:** Infection capacity of copepodites after knockdown with fragment 1 and 2 of LsPxtl-2 in nauplii and untreated controls

Gene	Fish	Total lice start	Chalimus II	PA I F	PA I M	PA II M	Total lice end	Percent survival	Statistics (# lice)
LsPxtl-1		0					0	0	
LsPxtl-2	1	60 control		11	2	10	23	38.33%	
	2	60 control		12		7	19	31.67%	
	3	60 control		10		11	21	35%	
	1	60 frag 1		6	1	13	20	33.33%	p = 0.042 (Frag 1 versus ctrl)
	2	60 frag 1	1	9	1	16	27	45%	
	3	60 frag 1		15	4	17	36	60%	
	1	60 frag 2		14	2	14	30	50%	p = 0.014 (Frag 2 versus ctrl)
	2	60 frag 2		21		9	30	50%	
	3	60 frag 2		9	2	16	27	45%	

Infection capacity of copepodites after knockdown with fragment 1 and 2 of LsPxtl-2 in nauplius stage and control copepodites

All LsPxtl-1-treated nauplius died after molting to copepodites. PAIF, pre-adult I female; PAIM, pre-adult I male; PAIIM, pre-adult II male; frag, fragment. Percent survival is for all stages included. Difference in sex distribution was analyzed using Chi-square analysis.

In contrast, infection with copepodites after knockdown of LsPxtl-2 in the nauplius stage did not show any negative effect on infection capacity; rather, the infection level (total number of lice attached) was higher in knockdown lice compared with untreated controls for Frag 1 (p = 0.042) and Frag 2 (p = 0.014) (Table 1). There was a skewing toward males for Frag1, however, not significantly different (p = 0.06) and lesser so for Frag2 (p = 0.83) (Figure 6).

### Antibody response and lice number after immunization trial

A vaccination and challenge trial was carried out to test the protective ability of these proteins as vaccine antigens. A single injection of a plasmid preparation was given to each fish, and a subgroup was boosted (n = 8) with a water-in-oil-based formulation of recombinant protein prepared from fraction with inclusion bodies. Initially we aimed to clone and express both variants but managed to prepare sufficient amount of protein for LsPxtl-2 only (Figure S4). An error in the original sequence obtained from the LiceBase for LsPxtl-1 was discovered causing a change in the length of the protein, preventing us from proceeding with making a protein vaccine for this variant. Thus, a boost injection of LsPxtl-2 antigen was given to a subgroup of the LsPxtl-1+2 primed group, termed LsPxtl-1+2B. The average number of lice (all stages included) (±SEM and 95% confidence interval [CI]) in different vaccine groups is shown in Table 2. The differences observed were analyzed using negative binomial regression, showing no difference in vaccinated groups compared with controls (Table 3).

**Table 2:** Average lice numbers in different vaccine groups

Vaccine groups	Mean lice	SEM	95% CI
Ctrl	20.057	1.227	17.63–22.48
LsPxtl-1	18.69	1.164	16.39–20.99
LsPxtl-2	21.135	1.243	16.11–22.11
LsPxtl-1+2	19.111	1.519	14.29–19.11
LsPxtl-1+2B	16.7	1.221	18.68–23.59

Demonstrate the average number of lice in the different vaccine groups (±SEM and 95% CI)

**Table 3:** Statistical analysis

	Coef	SE	Z	p value	95% CI
Ctrl	0.00	0.00			
LsPxtl-1	−0.07	0.08	−0.88	0.3781	[-0.23, 0.09]
LsPxtl-2	0.05	0.08	0.64	0.5207	[-0.11, 0.21]
LsPxtl-1+2	−0.05	0.10	−0.48	0.6339	[-0.25, 0.15]
LsPxtl-1+2B	−0.18	0.13	−1.43	0.1541	[-0.44, 0.07]
Lnalpha	−2.65	0.20			[-3.04, −2.25]
Alpha	0.07	0.01			[0.05, 0.11]
Intercept	3.00	0.06	51.06	0.0000	[2.88, 3.11]

Summary of statistical analysis, using negative binomial regression, of lice numbers where all vaccinated groups (as indicated) were compared with non-vaccinated controls (not shown)

P-values and 95% CI for the different groups are shown.

**Figure 7:**
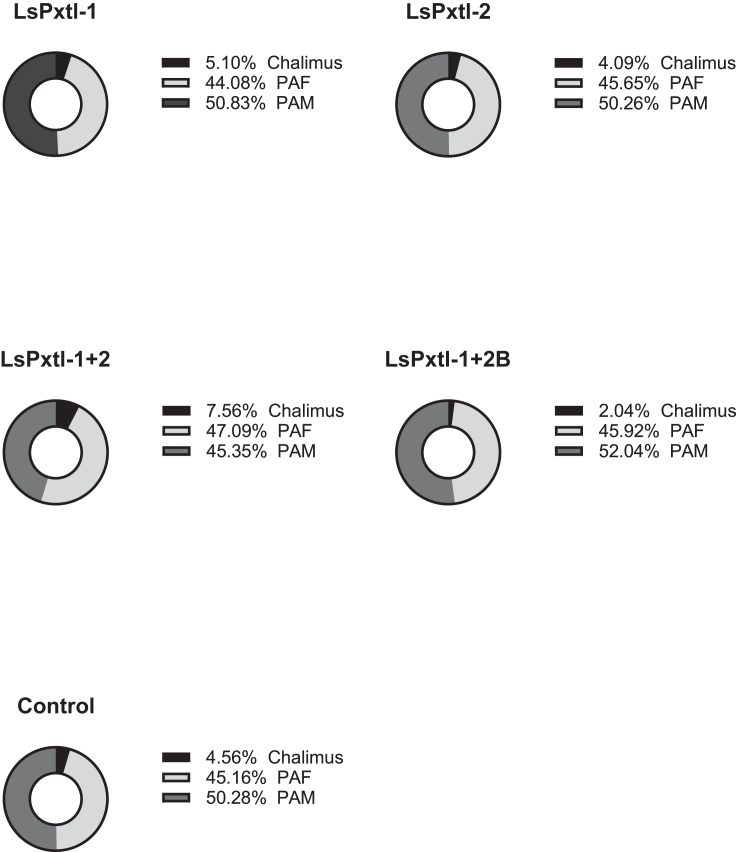
Percentage of the different life stages and gender from fish in different vaccine groups/controls at termination of vaccination and challenge trial Show the percentage of the different life stages and gender that was found on the fish in the different vaccine groups and control at termination of vaccination and challenge trial. PAF, pre-adult female; PAM, pre-adult male.

**Figure 8:**
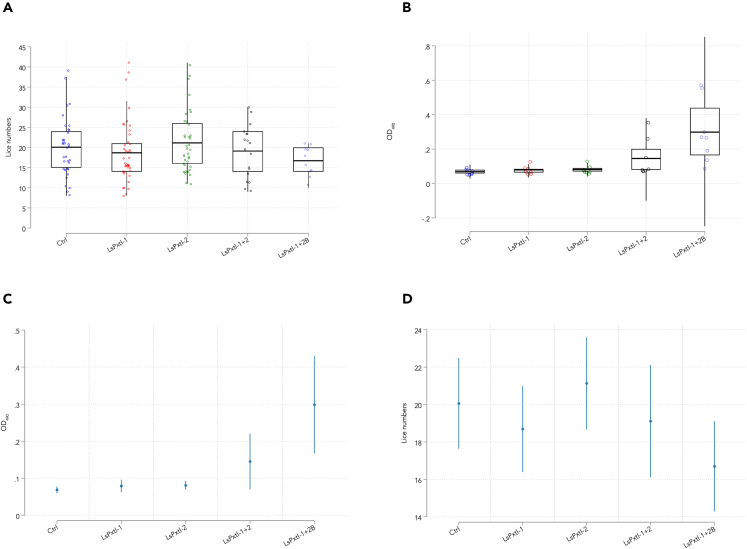
Graphs illustrate lice number and antibody response Atlantic salmon were immunized with three DNA vaccines, LsPxtl-1, LsPxtl-2, and LsPxtl-1+2, with a concentration of 20 μg/fish. Half of group LsPxtl-1+2 received a protein boost of LsPxtl-2 (50 μg/fish) named LsPxtl-1+2B. The control group was given PBS ip. The fish were infected with 60 copepodites/fish. Number of fish in each group: LsPxtl-1 n:42, LsPxtl-2 n:37, LsPxtl-1+2 n:8, LsPxtl-1+2B n:10, Ctrl n:35. (A and B) Number of lice collected from fish in each group illustrated with mean value with 95% CI. (B) The specific antibody response (OD) in serum of the control and the vaccinated groups and the effect of boosting using mean value with 25/75% quartile and 95% CI shown by whiskers (n = 8 per group). (C and D) OD values/antibody response for the different groups and corresponding lice numbers are shown of eight randomly chosen fish from each group, mean and 95% CI (whiskers). (D) Corresponding lice numbers in the different groups (same fish as in 8c), mean and 95% CI (whiskers).

At sampling, most lice had molted into the pre-adult 1 stage, and pre-adult II males started to appear (Figure 7). The lowest average number of lice was found in the LsPxtl-1+2B group (Figure 8A). However, it should be noted that the LsPxtl-1+2 and LsPxtl-1+2B groups had only 8 and 10 fish, respectively, compared with the other groups consisting of 35–42 fish (LsPxtl-1 n = 42, LsPxtl-2 n = 37, LsPxtl-1+2 n = 8, LsPxtl-1+2B n = 10, PBS n = 35).

Antibody responses were measured in vaccinated fish (Figure 8B). Only the LsPxtl-1+2 and LsPxtl-1+2B groups showed a significantly higher level of anti-lice antibodies than the non-vaccinated control group (p = 0.0341 and 0.0008, respectively). Further, combined plasmid injection of LsPxtl-1 and -2 increased antibody levels above controls (p = 0.005), and above single injection of LsPxtl-1 (p = 0.03) but not for LsPxtl-2 (p = 0.055). The antibody level in the LsPxtl-1+2B group was found to be significantly higher than the group receiving LsPxtl-1+2 encoding plasmids (p = 0.007, Figure 8B). We did not find a positive correlation between circulating antibody levels and lice numbers at individual level (rs = 0.1661, p = 0.3058) (Spearman’s correlation coefficient). Plotting antibodies and lice numbers by vaccine groups showed a trend toward falling lice numbers with increasing antibody levels (Figures 8C and 8D).

## Discussion

The present study employs a rational vaccine design starting from antigen selection through assessment of functional characteristics of candidate antigens by validating their importance for parasite development; this was followed by vaccine design and efficacy testing in a lab-scale infestation trial. We found that LsPxtl-1 is indispensable for viability of early developmental stages, whereas LsPxtl-2 did not affect viability. Injection of plasmids encoding the two peroxinectins elicited increased antibody responses when given as combined injections or as a plasmid-protein (LsPxtl-2) boost, but lice infestation postexperimental challenge was not significantly reduced.

The two peroxidase genes, LsPxtl-1 and 2, were classified as encoding peroxinectins based on common features with the family 3 peroxidase-cyclooxygenase superfamily.^47^ These features include a sequence with multiple Ca^2+^-binding domains in addition to several heme-binding domains,^47^ indicating that they are functional heme peroxidases. However, the different motifs revealed a slightly different pattern in terms of heme binding for LsPxtl-1; the first domain lacked features of a typical heme-binding domain, whereas the second domain contains these features. Presence of unfunctional domains is not uncommon in the dual oxidases and have been reported previously for members of the NOX/DUOX family.^48^ UniProt and the Redoxibase database were then used for diving further into potential functional traits, and a majority of hits for LsPxtl-1 and LsPxtl-2 showed high similarity to uncharacterized proteins, but a similarity hit to peroxinectins-like protein in *Penaeus monodon*, the black tiger shrimp (Id:3672), was found. This protein plays a role in cell adhesion, and similar findings are reported for *C. rogercresseyi* where two peroxinectins, PX-Cr1 and PX-Cr2, have been suggested to play a role in cell adhesion.^49^ Further, black tiger shrimp peroxinectins-like protein plays a role in activation of the prophenoloxidase system,^50^ which is thought to be a non-self-recognition system consisting of proteinases, pattern recognition proteins, and proteinase inhibitors, important in invertebrate immunity.^51^ Other functions known for peroxinectins are stimulation of phagocytosis and encapsulation.^52^^,^^53^ The latter function together with the heme-binding property may also be a mechanism for preventing heme toxicity,^54^ and of interest here is that LsPxtl-1 showed strong expression in the gut. In drosophila and mosquitos, knockdown of gut expressed dual oxidases documented their importance and involvement in immune responses and gut barrier functions.^35^^,^^36^

Here we found that knockdown of LsPxtl-1 in nauplii resulted in death shortly after molting into copepodites, indicating that the gene is crucial/indispensable for survival at this developmental stage. The abnormal growth in the intestinal region of these lice is an interesting observation and warrant further investigation in future studies because LsPxtl-1 was expressed in the gut. There were no phenotype changes observed for LsPxtl-2. On the contrary, after infecting salmon with the knockdown lice there was a significant increase in infection capacity for lice knocked down with frag 1 and 2 of LsPxtl-2. The reason behind this is not obvious to us but it could be related to the function of this peroxidase. In addition, more males were obtained from the fragment 2 knockdown lice. This finding is interesting and needs to be investigated in detail, as skewing the gender toward males can be used as a long-term strategy to reduce the lice population within the infected area. We also found that LsPxtl-1 and LsPxtl-2 are expressed in the ovaries and developing embryos of the eggstrings, and RNA sequencing showed variable expression within the same developmental stage, especially for LsPxtl-1, the latter would point to a role in lice development.^55^ Yet it is not known if they are directly involved in the reproduction or if they take part in production of certain developmental structures; the observed constitutive expression seen in all developmental stages supports the latter. Altogether, the underlying mechanisms are not deciphered, and more in-depth functional studies would be required to understand the functions of LsPxtl-1 and -2. Comparing the relative mRNA levels from the qPCR result with RNA-seq data shows some variations between the maturation stages. This may be due to different sample collection time points. RNA-seq data show a more detailed picture with several samples within each stage as well as sex differentiation from the chalimus stage, whereas this is not the case for the qPCR results. Studying the RNA-seq data shows large variation within some of the stages, thus making room for larger differences in the result when comparing the two methods used. However, the qPCR validates the general expression trend seen from the RNA sequencing. Aguiar et al. 2023 studied the comparability between the qPCR result and RNA-seq data and concluded that expression estimates had moderate correlation for some of the samples studied. Both technical and biological factors impact the results when using the two different techniques. Difference in normalization method, using different samples, collection point, storage, and storage length are among other factors that may cause variations in the results.^56^

Atlantic salmon mount a low and specific antibody response toward salmon louse after natural infestation.^23^ The concealed antigen strategy has earlier produced successful results for mammalian ectoparasites.^19^ In line with the rationale employed for parasite vaccines in higher vertebrates, the idea behind developing a vaccine against sea lice in salmon was to identify parasite target antigens important for development, survival, or the host-parasite interaction, as previously shown for *C. rogercresseyi* infestation in salmon.^57^ Here we targeted heme-peroxidases, and RNAi knockdown showed that LsPxtl-1 is more important for lice survival at early stages than LsPxtl-2. The reduction in lice number in vaccinees versus controls postchallenge was not statistically significant, concordant with previous studies employing recombinant vaccine technologies.^24^^,^^25^^,^^26^ We chose to immunize fish by plasmid injection, and as reported for DNA vaccines in salmonids, we found them to elicit low antibody levels.^58^^,^^59^ We included groups given a single injection of plasmid DNA (20 μg/fish) encoding either LsPxtl-1/-2 or a combination of the two plasmids (10 μg each). Earlier studies have shown that heterologous prime-boost vaccination can broaden the immune response, compared with a single injection or a homologous prime-boost vaccination that will elicit a more narrow immune response.^29^ Further, combination of a DNA vaccine and a recombinant protein vaccine has been found to induce neutralizing antibodies and T cell responses,^60^ and this also motivated us to use a DNA/recombinant protein combination. We found that a combination gave a significant rise in antibody response compared with controls, whereas plasmid injection with either LsPxtl-1 or -2 alone did not raise antibodies above background levels. From previous studies, it has been shown that plasmid vaccines elicit low antibody responses in salmon^59^; we aimed to produce recombinant protein of both LsPxtl-1 and -2 but were able to produce only LsPxtl-2 by recombinant expression in *Escherichia coli.* As expected, boosting the LsPxtl-1/-2 plasmid group gave a significant increase in circulating antibodies compared with combined plasmid injection, with a trend toward lower lice numbers with increasing antibody levels. One factor not tested in this study is the level of protein expression at site of injection.^61^ It has been shown that strong local antigen expression in membranes of muscle cells short time after injection correlates with improved protection, seen particularly for plasmid vaccines against viral hemorrhagic septicemia virus in trout.^62^ That said, a number of factors can impact immune responses, including size of the fish, concentration of antigen, type of adjuvant, nature of vaccine and administration route, boosting, and time between immunization and infestation. The observed trend giving lower lice numbers with increasing antibody levels is interesting and creates some optimism as regard to the possibility to employ prophylactic measures to control lice infestation. Adjusting vaccination modalities and also antigen doses may result in improved immunity. A larger sample size may have given more distinct trend. However, according to a method based on ANOVA, the E-value (Total number of animals−Total number of groups) for this study is adequate.^63^ An important obstacle when it comes to lice vaccines is that we poorly understand the initial recognition and attachment protocol employed by the copepodids, including the cross-talk between the parasite and the host at these early stages. Atlantic salmon is very sensitive to lice infestation compared with many other salmonid species, and the question is if vaccination can reprogram the molecular circuits of the immune response at early stage of infestation^64^ in such a way that it distorts the attachment process of the copepodids at this early stage. Overall, a rational approach toward vaccine development against sea lice infestation in salmon bears prospects for eliciting responses that impact on the infestation success of the parasite. We measured antibody responses generated after vaccination, but to what extent they would be a direct measure of protective responses or merely a proxy of a global immune response toward the parasite remains to be documented.

### Limitations of the study

The fish sample size used in the vaccination trial is small, however adequate for statistical analysis. The fish groups LsPxtl-1+2 and LsPxtl-1+2B were smaller than the other groups. Because we compared the groups together, we had to use the same sample size for all the groups. Boosting with only one variant, LsPxtl-2 protein.

## STAR★Methods

### Key resources table

**Table:** 

REAGENT or RESOURCE	SOURCE	IDENTIFIER
**Antibodies**		

Mouse anti-salmonid Ig-HRPO	Immunoprecise	Cat# CCOO6H
Anti-Digoxigenin (Mouse) HRP Conjugate	Revvity (PerkinElmer)	Cat# NEF832001EA

**Bacterial and virus strains**		

BL21(DE3) Competent Cells	ThermoFisher	Cat# EC0114
One Shot® TOP10 Competent Cells	Invitrogen (ThermoFisher)	Cat# C4040

**Biological samples**		

Salmon sperm DNA	Invitrogen ThermoFisher)	Cat# 15632011
Serum from Atlantic salmon	This paper	N/A

**Chemicals, peptides, and recombinant proteins**		

TRIzol™ Reagent	Invitrogen (ThermoFisher)	Cat# 15596026
Hydrogen peroxide	VWR	Cat# 1.07209.0250
Proteinase K	Merck	Cat# P2308
Triethanolamine	Merck	Cat# 90279
Acetic anhydride	Merck	Cat# 320102
Denhardt’s solution	ThermoFisher	Cat# 750018
Formamide	Merck	Cat# S4117
Dextran sulfate	Merck	Cat# D8906-5G
Bovine serum albumin	Merck	Cat# A3294
Streptavidin-Alkaline Phosphatase conjugate	Roche	Cat# 11089161001
Fast red substrate	Merck	Cat# F5146
GelCode™ Blue Safe Protein Stain	ThermoFisher	Cat# 24594
Montanide ISA 763 A VG	Seppic	N/A
TMB ELISA Substrate (High Sensitivity)	Abcam	Cat# ab171523
LsPxtl-2	This paper	N/A

**Critical commercial assays**		

RNeasy Plus Mini Kit	Qiagen	Cat# 74134
Transcriptor First Strand cDNA Synthesis Kit	Roche	Cat# 04 379 012 001
SMARTer™ RACE cDNA amplification kit	Clontech	Cat# 634923
SYBR™ Select Master Mix	Applied biosystems ThermoFisher scientific	Cat# 4472908
TSA plus biotin kit	PerkinElmer	Cat#NEL749A001KT
Q5 High-Fidelity DNA polymerase	NEB	Cat# M0491S
TOPO TA cloning kit	ThermoFisher	Cat# K4575J10
QIAprep Spin Miniprep Kit	Qiagen	Cat# 27104

**Deposited data**		

RNA-seq raw data	NCBI SRA	Accession: PRJNA413461
Lice and antibody levels raw data	This paper	https://data.mendeley.com/datasets/bh9cg59mw5/1

**Experimental models: Organisms/strains**		

Salmon lice*: Lepeophtheirus salmonis* (LsGulen)	Sea Lice Research Center	N/A
Atlantic salmon*: Salmo salar*	ILAB	https://ilab.no/

**Oligonucleotides**		

Primers for LsPxtl-1 and LsPxtl-2, see Table S5	This paper	N/A
Custom LNA mRNA Detection probes with double DIG for *In situ*, see Table S6	Qiagen	Cat# 339500
LsPxtl-1	NCBI	Accession number: OP649849
LsPxtl-2	NCBI	Accession number: OP649850

**Recombinant DNA**		

LsPxtl-1 plasmid	This paper	N/A
LsPxtl-2 plasmid	This paper	N/A

**Software and algorithms**		

NCBI GenBank	Altschul et al.^65^	BLAST: Basic Local Alignment Search Tool (nih.gov)
Expasy	Duvaud et al.^66^	Translate - SIB Swiss Institute of Bioinformatics | Expasy
Prosite	Sigrist et al.^44^	https://prosite.expasy.org/
Interpro	Blum et al.^45^	https://www.ebi.ac.uk/interpro/
Uniprot	Consortium^42^	UniProt
Redoxibase	Savelli et al.^43^	RedOxiBase: Home page (inra.fr)
NGPhylogeny.fr	Lemoine et al.^46^	https://ngphylogeny.fr/
CLC Workbench 692	Qiagen	https://digitalinsights.qiagen.com/products-overview/discovery-insights-portfolio/analysis-and-visualization/qiagen-clc-genomics-workbench/
GraphPad Prism 9.1.0	GraphPad	https://www.graphpad.com/
Stata17	Stata	https://www.stata.com/

### Resource availability

#### Lead contact

Further information and requests for resources and reagents should be directed to and will be fulfilled by the lead contact, Øystein Evensen (oystein.evensen@nmbu.no).

#### Materials availability

This study did not generate new unique reagents.

### Experimental model and study participant details

#### Salmon lice (*Lepeophtheirus salmonis*) and Atlantic salmon (*Salmo salar* L.)

The lice used in the experiments (LsGulen) was maintained on Atlantic salmon as previously described^67^ at the lice lab, Sea Lice Research Center, University of Bergen, Norway. Salmon used in the experiments were purchased from ILAB and were fed a commercial diet and reared in sea water with a salinity of 34.5 ppt and a temperature of 9 ± 0.5°C if not otherwise mentioned. Eggs, nauplii, and copepodids maintained off the host were kept in incubators with continuous flow-through of seawater from the same supply as the fish tanks.^67^

### Method details

#### RNA extraction (isolation) and cDNA synthesis

Adult lice, one female and two males combined, were homogenized with FastPrep24 machine (MP Biomedicals, California, USA) using stainless beads. The RNA was then extracted from the homogenate using a combination of Trizol (ThermoFisher scientific, Massachusetts, USA) and RNeasy Plus Mini Kit (Qiagen, Hilden, Germany). Briefly, chloroform (0.2 ml) was added to lice homogenate prepared using 1ml Trizol for phase separation. The aqueous phase was then used for RNA isolation using RNeasy Plus mini kit following the manufacturer’s protocol.^68^ After extraction, the RNA concentrations were measured by spectrophotometry using Nanodrop ND1000 (Thermo Scientific, Massachusetts, USA). cDNA was synthesized from RNA using Transcriptor First Strand cDNA Synthesis Kit (Roche, Basel, Switzerland) following manufactures protocol.

#### Identification and characterization of two salmon louse peroxidases, LsPxtl-1 and -2

First, an initial search for peroxidase in salmon louse was performed in the NCBI GenBank,^65^ and two partial sequences of peroxidase-like genes were found. The first gene (accession no.: XM_040724286) did not show any resemblance to any characterized peroxidases, while the second gene (accession no.: XM_040714389) was defined as eosinophil peroxidase-like protein. The sequences were then identified in the salmon louse genome as EMLSAT000000009839 and EMLSAT00000004874,^39^ and RACE PCR were performed to obtain the two mRNA sequences using primers listed in Table S5 and SMARTer™ RACE cDNA amplification kit (Clontech, California, USA).^40^ Subsequently, a partial sequence of LsPxtl-1 and a full sequence of LsPxtl-2 were obtained. In order to characterize the two genes, the sequences were translated using Expasy,^66^ and the protein sequences were blasted and domains identified in Prosite^44^ while only additional domains prediction was performed in Interpro.^45^

#### Phylogenetic analysis

To perform phylogenetic analysis, both nucleotide and protein sequences obtained for the two genes were blasted in Uniprot,^42^ and the top 25 closely related sequences were selected for further analysis. A similar blast search was performed in the more specialized peroxidase database, Redoxibase,^43^ but this time only for the protein sequences due to the high similarity obtained between the nucleotide and protein blast in Uniprot. Subsequently a phylogenetic analysis was performed using the top 25 closely related sequences obtained from both databases using NGPhylogeny.fr.^46^^,^^69^

#### Ontogenetic analysis

RNA isolation and cDNA synthesis of salmon louse for ontogenetic analysis has already been described elsewhere.^40^^,^^70^ The amount of RNA used for making cDNA was 1 μg. Primers used for qPCR detecting the two peroxidase genes are described in Table S5. Previously designed and validated primers for elongation factor 1α (eEF1α) was used as reference gene.^71^ SYBR Select master mix (ThermoFisher scientific, Massachusetts, USA) was used for the real-time RT-PCR following manufactures recommendations. Briefly, a duplicate of samples consisting of a 10 μl reaction mix with 1x master mix, 2 μl diluted cDNA and 500 nm of forward and reverse primers were run under standard conditions (50°C for 2 min, 95°C for 2 min, and 40 cycles of 95°C for 15 s and 60°C for 1 min, followed by a melt curve analysis at 60–95 on the Applied Biosystems 7500 REAL-TIME PCR System.^40^ In order to obtain a normalized expression, the 2-ΔΔCt calculation was used using Ct value obtained for the eEF1α and LsPxtl-1 and -2 expression in eggstring as basis for calculation of peroxidase expression in later development stages.

#### RNA sequencing

The detailed RNA sequencing procedure has already been published,^39^ and the two salmon louse heme peroxidases (LsPxtl-1 and LsPxtl-2) were obtained from the raw sequences from the referred study and further analysis here. In brief, the Ls1a strain^67^ of *L. salmonis salmonis* that was inbred for 27 generations was sequenced to 181-fold assembly coverage in a hybrid approach using Illumina, 454 pyrosequencing. Further, expression analysis of LsPxtl-1 and LsPxtl-2 in different development stages including copepodids, chalimus, pre-adult and adult stages was carried out on raw data from a previous study.^55^

#### *In situ* hybridization

Custom LNA mRNA Detection probes with double DIG label were designed and produced by Qiagen for both genes (Table S6). A formalin fixed samples of adult female salmon lice with egg strings were embedded in paraffin under RNase free conditions using standard procedures and longitudinal sections of about 3 μm were mounted onto poly-L-lysine-coated slides (Thermo scientific). The sections were dewaxed and rehydrated using xylene and three different series of ethanol dilutions (100% x2, 96%, 70%). Thereafter, the sections were incubated in methanol with 0.1% hydrogen peroxide (VWR, Pennsylvania, USA) for 25 minutes to stop endogen peroxidase activity. Next, the sections were incubated with Proteinase K (40 μg/ml) (Merck, Darmstadt, Germany) in diethyl pyrocarbonate (DEPC) treated tris-EDTA (pH 7.4) (TE-buffer) for permeabilization at 37°C for 30 minutes. Then 4% formaldehyde in PBS was added for 6 minutes at 4°C to stop proteinase K digestion followed by two times washing in DEPC treated PBS for 5 minutes. Sections were subsequently acetylated with triethanolamine (Merck) containing acetic anhydride (Merck) for 5 minutes at room temperature (RT). The sections were thereafter incubated with acetic anhydride for 5 more minutes before being washed two times in DEPC PBS for 5 minutes.

For prehybridization, the sections were covered with 250 μl prehybridization buffer (20xSSC, Denhardt’s solution (ThermoFisher), formamide (Merck), salmon sperm DNA (ThermoFisher) and DEPC water) and further protected with cover film. This was followed by incubation at 42°C for 1 hour in a StatSpin Hybridizer (Dako, Santa Clara, USA). Sections were further covered with 500 μl hybridization buffer (20xSSC, Denhardt’s solution, formamide, salmon sperm DNA, Dextran sulphate (Merck) and DEPC water) containing the double DIG labeled DNA probes and the slides were covered with film and placed in the hybridizer at 84°C for 10 minutes. Sections were immediately chilled on ice for 2 minutes and further incubated at 42°C overnight. This was followed by another washing steps using 0.5xSSC in DEPC-treated water first 2 × 15 min at 55°C and then one time for 10 min at RT before rinsing the sections in PBS.

The detection procedure was initiated by blocking the sections with 5% bovine serum albumin (BSA) in PBS for 30 min at RT. Next, mouse anti-DIG-Horseradish peroxidase (HRP) labeled antibodies diluted 1:1000 in 2.5% BSA in PBS were added for 1 hour at RT. Amplification of the signal was carried out with TSA plus biotin (Perkin Elmer) and later Streptavidin-Alkaline Phosphatase conjugate (Roche) diluted 1:100 in 2.5% BSA in PBS added to the sections for 30 min in RT. Washing with PBS 3 × 5 min were carried out between each step. The signal was developed by adding a filtered fast red substrate (Merck) for 5 min followed by washing in distilled water. Finally, sections were counterstained with Mayer`s hematoxylin for 10 seconds followed by washing in running tap water for 10 minutes and then mounted in Aquatex (Merck) before being studied under the microscope.

#### RNA interference in nauplius and pre-adult

Knock down experiments were carried out in nauplius and preadult salmon lice using standardized protocols.^72^^,^^73^ Two fragments from each sequence were used for RNAi in nauplius I. The RNAi trial for LsPxtl-1 and LsPxtl-2 consisted of three groups for each (total of 6 groups). The first group of lice received dsRNA with fragment 1, the second group fragment 2, and the last group was control lice treated with dsRNA CPY (cod trypsin) control gene. Nauplius I were exposed to 1.5 μg dsRNA in a treatment bath right before initiation of molting to nauplius II. Exuvia from all the animals were observed under the microscope the next day to verify the finalization of the molting process before terminating the treatment. A replicate of the experiment was carried out for both genes. Treated lice (60 lice/fish) were, after molting to copepodids, allowed to infest Atlantic salmon kept in a single tank system, one salmon for each group. Primers used for dsRNA are listed in Table S5.

For the experiment performed in preadult, female lice were injected into the cephalothorax with approximately 600 ng dsRNA. One dsRNA fragment was injected for LsPxtl-1 while two were used for LsPxtl-2. The lice were then placed on Atlantic salmon and three different experimental groups were created. The first group received lice treated with LsPxtl-1 fragment, group 2 received lice treated with fragment 1 of LsPxtl-2, and the last group received lice treated with fragment 2 of LsPxtl-2. Three Atlantic salmon were used for each fragment/group and 20 lice (1:1 sex ratio) were placed on each fish. In addition, three fish receiving lice injected with the control fragment dsCPY were included. The different preparation steps and the experimental procedure used has been published in detail elsewhere.^72^ Experiments were terminated after one month for copepodites and 42 days for pre-adult, and remaining lice were counted and collected from the fish to be further analyzed.

#### DNA and protein vaccine preparation

PCR amplification of the sequence were carried out using Q5 High-Fidelity DNA polymerase (NEB) and primers listed in Table S5 following the protocol recommended by the manufacture with slightly modified cycling condition (98°C for 30 sec, (98°C for 20 sec, 68°C for 1,30 min, 72°C for 1 min) ×40°C and 72°C for 10 min). Next the PCR products were inserted into TOPO vector using TOPO TA cloning kit (Thermo Fisher) following the manufactures protocol, and subsequently transformed into E. coli TOP 10 competent cells (Thermo Fisher). The transformed bacteria were then plated onto a LB agar media containing 100 μg/ml Ampicillin. Positive clones were collected and verified by PCR and sent to GATC (Eurofins) for sequencing. Plasmids from positive clones were subsequently extracted and used for subcloning. Sequences for the primers used for PCR, cloning and sequencing are listed in Table S5. For subcloning, PCR products were obtained using plasmid containing the two genes described above as templates. Primers were designed to include restriction sites HindIII and Bam (Table S5), in order to allow insertion into the plasmid vectors pcDNA3.1A and pET32c which were used for preparation of DNA and protein vaccines respectively. Clones were verified by sequencing and one clone containing the right insert was used to transform top 10 competent cells for DNA vaccine and BL21 (Thermo Fisher) for protein preparation. As we were unable to obtain the protein for LsPxtl-1 using primers listed in Table S5, further preparation for protein vaccine was only carried out for LsPxtl-2.

To prepare the vaccines, bacteria were grown in LB broth containing 100 μg/ml Ampicillin. For the DNA vaccines, plasmid DNA was purified using QIAprep Spin Miniprep Kit (Qiagen) following protocol described by the manufacturer and concentrations were measured by Nanodrop ND1000 (Thermo Scientific). The concentrations were then adjusted by dilution in PBS and made ready for injection. For the protein vaccine, purification of protein was performed by centrifuging the cultures followed by resuspension in PBS and sonication (cycle 0.5, amplitude 40 for 20 min with breaks). Inclusion body fraction was obtained by centrifugation at 10 000–12 000 g for 10 min and resuspended in PBS before concentrations were evaluated by Bio-Rad protein assay and a standard dilution using bovine serum albumin. The expression of proteins with correct size were verified by SDS-PAGE protein gel using Gelcode Blue Safe Protein Stain (Thermofisher Scientific) (Figure S4). The adjuvant Montanide ISA 763 A VG (Seppic), 7,4 ml, was used for the recombinant protein vaccine and was mixed with 2,6 ml protein antigen (70:30 w/w).

#### Immunization and sampling

Three groups of Atlantic salmon (average weight 68 gram) were injected intramuscularly (i.m) in the abaxial muscle below the dorsal fin with plasmids. The first group received an injection with LsPxtl-1, the second group with LsPxtl-2, and the third group a combination of LsPxtl-1 and LsPxtl-2 (LsPxtl-1+2). Group 4 as injected with PBS intraperitoneally (i.p.) and served as control, and only i.p. route was selected for controls (PBS will not induce responses independent of injection route). Half of the fish in the LsPxtl-1+2 group received an i.p. injection with LsPxtl-2 protein, administered 40 days post primary immunization and availability of fish prevented increasing fish number in these groups. The fish were exposed to 24-hour light to induce smoltification for 504 degree-days after immunization and then transferred to sea water within a few days. All fish groups were kept together (common garden experiment) in one circular tank (3 m in diameter) with a water temperature at 12°C throughout the experiment. Challenge was carried out in stagnant water with oxygen added during the challenge. After 30 min, water flow was resumed. Infestation with salmon lice (60 copepodites/fish) was carried out 3 months after primary immunization and the trial was terminated 18 days after challenge. Blood samples were collected the day of termination (Figure S5) in vacutainer (BD), and serum separated from whole blood by centrifugation at 2500 rpm for 10 min at 4°C. An overview of the different vaccine groups, the infestation dose and the timeline for the trial is provided in Table S7 and Figure S5.

#### Enzyme-linked immunosorbent assay (ELISA) and validation

ELISA was performed using salmon louse homogenates prepared from formalin fixed lice. Briefly different life stages of salmon lice were preserved in 10% phosphate-buffered formalin, including copepodites, preadults and adults of both genders. Prior to further processing, the lice were washed three times in sterile PBS. Afterwards, PBS containing protease inhibitor was added to the lice to prevent protein degradation and lice were homogenized using FastPrep-24 (MP Biomedicals). Homogenate was cleared by centrifugation and the protein concentration (in the supernatant) was determined. This homogenate (0.44 mg/ml) was aliquoted into small portions and kept at −80°C until use. The ELISA plates (Thermo Fisher Scintific-Nunclon) were coated using prepared homogenates diluted 1:1000 in bicarbonate buffer (0.795 g Na_2_CO_3_ + 1.465 g NaHCO_3_ + dH_2_O up to 500 ml) and incubated at 4°C overnight. The plates were then washed three times with 250 μl washing buffer (PBST) using Biochrom Asys Atlantis automated 96 plate washing machine (Biochrom, Cambridge, United Kingdom). Then, 250 μl/well of blocking buffer (5% fat free dry milk (Bio-Rad, California, USA) in PBS with 0,05% Tween (PBST)) was added to the wells, and the plates were incubated at room temperature for 2 hours. After 3x wash as described above, the plates were incubated with 100 μl of salmon serum diluted 1:400 in diluent buffer (1% fat free dry milk in PBST) at 4°C overnight. The plates were then washed as above before adding 100 μl of HRP labeled antibody mouse anti-salmonid immunoglobulin (Immunoprecise, 1 mg/ml, Victoria, Canada) diluted 1:2000 to the wells and incubated for 1 hour in RT. Afterwards, the plates were washed and 50 μl of TMB substrate solution (Abcam, Cambridge, United Kingdom) was added to each well and incubated for maximum 10 minutes in the dark at RT. The reaction was stopped by adding 50 μl 1M HCl. Finally, Tecan Genios spectrophotometer (Männedorf, Switzerland) was used for measuring absorbance at 450 nm.

### Quantification and statistical analysis

#### Statistics and software

CLC workbench was used to aligned nucleotide sequences and the amino acid sequences for both genes. Statistics were performed using GraphPad for evaluation of the ontogenetic analysis. Statistical assessment of effect of vaccination and antibody levels was done by using Stata17. Differences in lice numbers between vaccines and controls or for RNAi treated lice (Frag1 and Frag2) post infection were analyzed using negative binomial regression analysis. Non-parametric Kruskal-Wallis test was used with Dunn’s multiple comparison post hoc test to test the difference in weight and length between the vaccine groups. Difference in sex distribution between Frag1, Frag2 and controls post infection was done by Chi-square analysis (Stata17). Graphical abstract was created with BioRender.com.

### Additional resources

The experiments performed on live animals were authorized by the Food Safety Authority in Norway and following the Norwegian animal well-fare legislation (approval number ID8589).

## Data Availability

• The nucleotide sequence for LsPxtl-1 and LsPxtl-2 can be found in the NCBI genebank and are publicly available as of the date of publication. Accession numbers are listed in the key resources table. • RNA-seq data are accessible through NCBI SRA genebank. Accession number are listed in key resources table • Any additional information required to reanalyze the data reported in this paper is available from the lead contact upon request. • The datasets generated and/or analyzed during the current study are available at repository https://data.mendeley.com/datasets/bh9cg59mw5/1 • This paper does not report original code.
