# Supplementary material for: Identification and characterization of two salmon louse heme peroxidases and their potential as vaccine antigens
Source: iScience. 2023 Sep 21;26(10):107991. doi: 10.1016/j.isci.2023.107991 (PMC10579435; doi:10.1016/j.isci.2023.107991)
Supplement: Document S1. Figures S1–S5 and Tables S1–S7 [file mmc1.pdf]

iScience, Volume *26*

Supplemental information

Identification and characterization of two salmon louse heme peroxidases and their potential as vaccine antigens

Elisabeth Gislefoss, Amr Ahmed Abdelrahim Gamil, Aina-Cathrine Øvergård, and Øystein Evensen

Figure S1. DNA and protein sequence for LsPxtl-1, related to Figure 1A. A) DNA sequence. B) Protein sequence.

A)
atgaggatggagtgggtactcctcgtgagtcctctattttggaagaaaaaaaacttcctgatgcgtcaactgtctcatctaaacttgtttggaca
cataacaatcctcatgaacacatcactgccattgcagctacatgggggagaatttgttgctcatgatatcagctatactctccctctatccggat
ttgagcagtgttgcgactccatgtttcgaaaagaaaatgccatggaatgcttccccattgtcaatgatcaaacagtttgccaagagtatgttcg
ctctgccattggtctcaagcctggttgtgttctgggaccaagggagcaaatgaactttgccactgcgtttttagatggctccggaatttatggatc
tactcccaagtctggtgaagataggaggaaattggaaggtggattgcttaaaatgggagatcctgaaggcaatgttcttcctttggataaga
ataatcccaattgtcgtcctaatttggatgggcatgaatgttttaaatcaggggatgaaagagttaatcacaacgcgggattagcagtccttca
tactttattggcaaaagaacacaatcgagtagctcgaagccttgagggagtgaaccctcattgggacgaagagacactataccaagagg
ctcgacgcatagtatcagcagaaattcaacacataacctacaatgaattcctcccagccattttgggtgaagtcctaatgaagacttttgacct
tgagccaaaatcaaatggatataatatggaatatgatgaagaacttcctgtcactactcttaactctgttggtaatgcaatccttccattcgtgct
ctcacttttcccacctaaattgaacttcttcaatagtaagggaaagcaaacgggccaagttcctttcaattccacctattgggctcctttccacgc
gaaagataaaaaactcatttctgaaatacttctgggactatcacgaacaccagcccaaaaggctgatcttgttatggctagggacaccaag
atttatttaaatcaggatctcgttgcaaacattattcacagaggacgtgatcatggtttacctacttatacgcaagtccgagagacttgtggccttt
ctcccgttcgaaattttgaggagctaagtaacactgtggacaaggaagtgataggacttttggagacaacctatgagcacgtaagtgatgtg
gatctcttcattggaggtctagcagagcggcctctttccggtggagttgttggccctacctttggttgtctgttgggtcaacagtttcaaatattgaa
aaaaggagatcgcttttggtacgaaaataacattcctccctccgcttacggaaaggatcaactcttagaacttcgtaaggtcacactggcatc
tttgatttgttccaactttaaggacattcatgaagtacagcccgaggtttttctcaacagtgatccttatttgaattctccagttcattgtgatatttttcc
gcaaattaactttcaacaatggaaaactgatagtcctgcccttcaagtccccgaggctattttaattcaaacacttgaaaaaggaacacaag
gtcttgtcaagcggaggaaagaggagtacatgctttgggaacataacatgggagcaaatcctcagtcctcattgggaacagcattcgctttt
agccgtcccaacaaagttgctcttaaattgtcgaatacctctcttctcctcgaatatacatccagagtctttatcaactcccttcaagagcctgaa
aataatcgaatcaaacgtcaagtaactggaaatagtgactccattgatgtaaatattgatgactttgcggatggtttaaccaacattgacgtta
cacgggtgatccccacaaatgctccactggaggtttgtccatttgatgaaaacagtcttccttgtgaccacacccataaatatagaactatga
atggatggtgtaataacctcaataatcctcagtacgggaaaagtgtcaccccacttgttcgatttctatcagcaaaatatgatgacgctatatct
cgtccaaggttcaggtctgctacgggtaagagcctcccatctcctcgaatggtgtctatagtggtacatgctgatgtttcccatctacatggacg
atacactcttatgctcatgcaatttggacaatttttggatcatgacattacgctcactcccgtcaataaaggcttccctgattccattttggattgtcg
tggttgtgactctgcgcgaactgttcatcctgagtgttggcctatttctgtcccaaaaaatgatccctattttcctgcattaaataccacttctggaa
gagcacattgtattgcatttacaagatccctacctggtcagcaacgacttggcccacgagagcaagttaaccaaaatacggctttcattgatg
catctatgatttatgggaatgacaaatgtgactctattgatttacgacaaaacatcgatggtaggctgaacactacactacccagtcatggaa
gaggcaaacatcttatgcccaaggatagttccaataaggaatgtaaagcggcttcaggattttgtttcaaaggaggagatgctagagcctct
gagcagcctggacttgctgctttacatactatttttctcagggaacataatcgtctcgtcaccaagctgaaaagagtcaatcctcattggagtg
atgacgaactttattatgagggaaggcatattttatcagctgtgaatcaacacatcacttataatgagttccttcctcggatcattggttggaatta
tatgaacttgtataatcttcgagttcaaacccaaggatattcttcagactatgaatctacctgtaatcctggcatattcaatgaatttgccacagct
gccttcagatttggtcattcactcattcgtcccatgcttacccgtatgtctgctgattggaaggaaaagaaaaagcatattcgtctacgtgatgg
atttttcaacccagatatgttatatcaagaaacaatgattgatgaagttatcagaggtctagttgctacgcctatggagaaccaggatcagttta
tatccggagaaataaccaaccatttgtttgaagaaaaaaaaaaattccattttctggtcttga

B)
MRMEWVLLVSPLFWKKKNFLMRQLSHLNLFGHITILMNTSLPLQLHGGEFVAHDISYTLPLSGFEQ
CCDSMFRKENAMECFPIVNDQTVCQEYVRSAIGLKPGCVLGPREQMNFATAFLDGSGIYGSTPKS
GEDRRKLEGGLLKMGDPEGNVLPLDKNNPNCRPNLDGHECFKSGDERVNHNAGLAVLHTLLAKE
HNRVARSLEGVNPHWDEETLYQEARRIVSAEIQHITYNEFLPAILGEVLMKTFDLEPKSNGYNMEY
DEELPVTTLNSVGNAILPFVLSLFPPKLNFFNSKGKQTGQVPFNSTYWAPFHAKDKKLISEILLGLSR
TPAQKADLVMARDTKIYLNQDLVANIIHRGRDHGLPTYTQVRETCGLSPVRNFEELSNTVDKEVIGL
LETTYEHVSDVDLFIGGLAERPLSGGVVGPTFGCLLGQQFQILKKGDRFWYENNIPPSAYGKDQLL
ELRKVTLASLICSNFKDIHEVQPEVFLNSDPYLNSPVHCDIFPQINFQQWKTDSPALQVPEAILIQTL
EKGTQGLVKRRKEEYMLWEHNMGANPQSSLGTAFAFSRPNKVALKLSNTSLLLEYTSRVFINSLQ
EPENNRIKRQVTGNSDSIDVNIDDFADGLTNIDVTRVIPTNAPLEVCPFDENSLPCDHTHKYRTMNG
WCNNLNNPQYGKSVTPLVRFLSAKYDDAISRPRFRSATGKSLPSPRMVSIVVHADVSHLHGRYTL
MLMQFGQFLDHDITLTPVNKGFPDSILDCRGCDSARTVHPECWPISVPKNDPYFPALNTTSGRAH
CIAFTRSLPGQQRLGPREQVNQNTAFIDASMIYGNDKCDSIDLRQNIDGRLNTTLPSHGRGKHLMP
KDSSNKECKAASGFCFKGGDARASEQPGLAALHTIFLREHNRLVTKLKRVNPHWSDDELYYEGRH
ILSAVNQHITYNEFLPRIIGWNYMNLYNLRVQTQGYSSDYESTCNPGIFNEFATAAFRFGHSLIRPM
LTRMSADWKEKKKHIRLRDGFFNPDMLYQETMIDEVIRGLVATPMENQDQFISGEITNHLFEEKKN
SIFWS

Figure S2. DNA and protein sequence for LsPxtl-2, related to Figure 1B. A) DNA sequence. B) Protein sequence.

A)
atgtttggagttaaggaacaaatgtctttactatgcatatgtctctatattgttaaagtctacggagaaggctgtccatttgcatcttcggctaatga
tgaaaaaaaacttttagatcaggcaaatgtcaaggatgtagactatcataaattgcttcagaaaggaagacaagtaacgccaaattctctc
aaaatgtctcgaagaaatatatttgttgacaaaacggtaaacagattggtgaaaaaaggttattcaagagactctgcacatcaaaaggtaa
tccaactaataaaagatattgagtatgagcttgaggaatgcaaaggaacaatgccagattgtgacccaaacatgaagtatcggatgatga
atggacaatgtaataaccctgttaatcctttgtttgggtctcagagatacaggctgcaacgtttttttgccacctaaatacgaggatgcggaatca
aacccaactgaaaacagtcaatttaaagctagagagggatgtatttctagtggtcctctaccaaatgcaagaaaagtcagttcaacagtgc
atactgatgtggatgttccttcgacgattgcgtctactatggtaagccaatggggaatgtttgttgcccatgatttgtcctttgttcccaattcccac
gaggctaaatgctgtgaaggagcaaaggatgacagatgccttccgattagaatatatgaaaatgaccttttctattcaaatttcaatgtgacat
gtttgaatttttatcgatctgtcagattttgcagttcaactccaagggaacagtataatgaaattacttcctttattgatgcttccaatgtctatggctc
aagcgacgaggaattattgctactcaaggaagaaaattcttacttaatgaagagtacgctgactgatgatggaaaagaactccttcccaaa
gatggtaaaggtagttttgtgggaggagacgctagagctcttctaaccactggtttggcttcagttcataccttattcgttcgggaacataatcg
aatatcaaggaaacttgctcagatgaatccttcatacacaaacgaaactgtttttcaagaaactcggcgaatattgggagcaatgatgcaa
aatattgtttatagtgaatttcttccgacgttattaggggaaaaaagcatgaatgagtctggcttaaacttaaaatacccctcgagttatcatgaa
gaaattaatccaactgtcgcctctgttttcgttgctgctgcatacagatttggccattccatgattcaaaggaatgtgaatcgattggctataaaa
aaatatagctatagcagtaattattccttatttaaggactttttaatacggcaaattacgaggataggaatgggctaggaatagaagaaatttt
atatgggttgacctatttcccagcacaatctgcagaccgttttgttgtaaaagaagtaactaattatttgttcaataatggaaagccgtatggag
cagatcttgaagcaaaaacgattttgaggggtcgagatcatggattaccctcttatggagattggcgagaattctgtggcttagaaccactttg
tgactggaacaataaaccaacagaaatttcagaaaatatctggcttcttctcaaagagttatattcaagtcctaatgacatcgatattcacaca
gcaggacttgcagaaactccaattccacactctgtcttggggcccaccaataattgcataattggacgtcaatttaccgctctaaaagatgga
gatcggttctttttcacacacgatggtgaagctggttcctttaccccaaaacaagtcaaggcacttcgtcaaacatccctctcacgtgtcatgtg
tgataacacagggattgataagataagtgtaaatgttttcaggaccgattcccaatttgtaccttgttcaagtctccctgagttgaatcttaatttat
tctaa

B)
MFGVKEQMSLLCICLYIVKVYGEGCPFASSANDEKKLLDQANVKDVDYHKLLQKGRQVTPNSLKM
SRRNIFVDKTVNRLVKKGYSRDSAHQKVIQLIKDIEYELEECKGTMPDCDPNMKYRMMNGQCNNP
VNPLFGSQRYRLQRFLPPKYEDAESNPTENSQFKAREGCISSGPLPNARKVSSTVHTDVDVPSTIA
STMVSQWGMFVAHDLSFVPNSHEAKCCEGAKDDRCLPIRIYENDLFYSNFNVTCLNFYRSVRFCS
STPREQYNEITSFIDASNVYGSSDEELLLLKEENSYLMKSTLTDDGKELLPKDGKGSFVGGDARALL
TTGLASVHTLFVREHNRISRKLAQMNPSYTNETVFQETRRILGAMMQNIVYSEFLPTLLGEKSMNE
SGLNLKYPSSYHEEINPTVASVFVAAAYRFGHSMIQRNVNRLAIKKYSYSSNYSLFKDFFNTANYED
RNGLGIEEILYGLTYFPAQSADRFVVKEVTNYLFNNGKPYGADLEAKTILRGRDHGLPSYGDWREF
CGLEPLCDWNNKPTEISENIWLLLKELYSSPNDIDIHTAGLAETPIPHSVLGPTNNCIIGRQFTALKD
GDRFFFTHDGEAGSFTPKQVKALRQTSLSRVMCDNTGIDKISVNVFRTDSQFVPCSSLPELNLNLF

Figure S3. Phylogenetic tree illustrates the relations between the identified proteins and proteins of different species using the Uniprot database[1], related to Figure 2. A) Protein sequence of LsPxtl-1. B) Protein sequence of LsPxtl-2. The sequences were blasted in the Uniprot database and top 25 proteins was added to the tree based on highest identity and E-value. UP: Uncharacterized protein, CP: Chorion peroxidase, CPL: Chorion peroxidase-like protein, CPP: Chorion peroxidase precursor, PD: Peroxidasin, PDL: Peroxidasin-like protein, PDfrag: Peroxidasin fragment, Clip: Clip domain-containing protein, TPL: Thyroid peroxidase-like. The illustration tree is made by NGPhylogeny.fr [2]

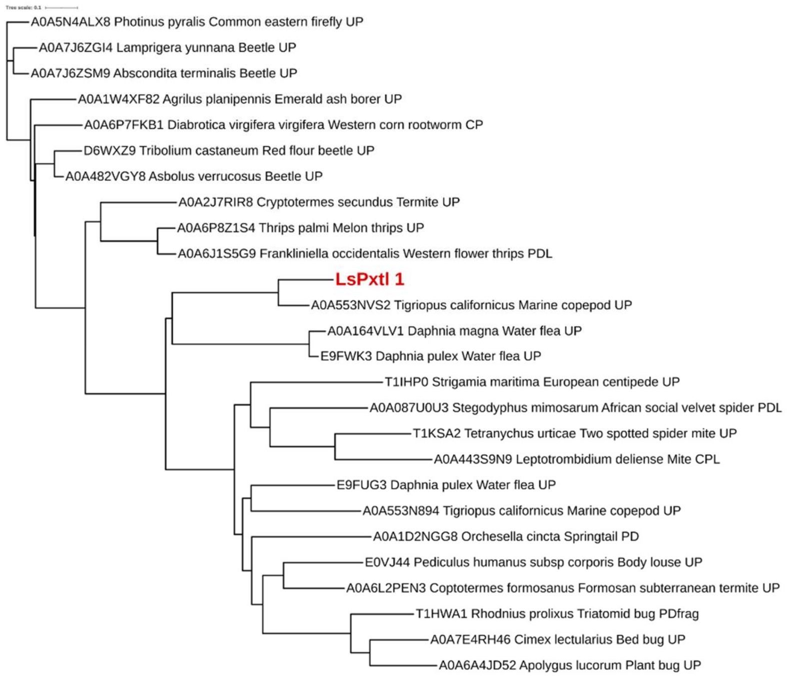

A)

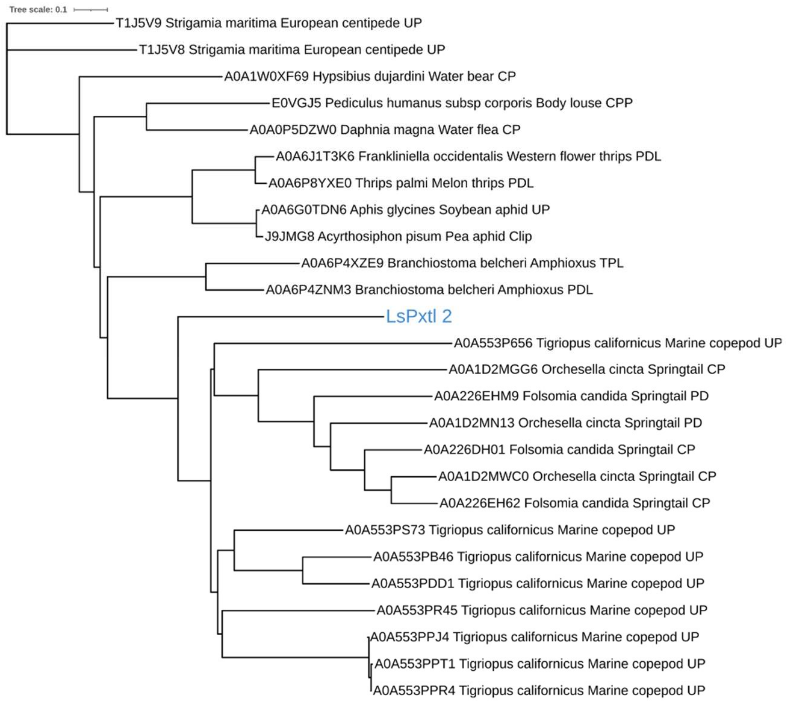

B)

Figure S4. Protein validation of LsPxtl-2, related to STAR Methods. Protein gel stain of LsPxtl-2. 1: Seeblue ladder, 2: Precision blue ladder. 3: Induced LsPxtl-2 Inclusion body fractions. 4: Non-induced inclusion body fraction. 5. Supernatant.

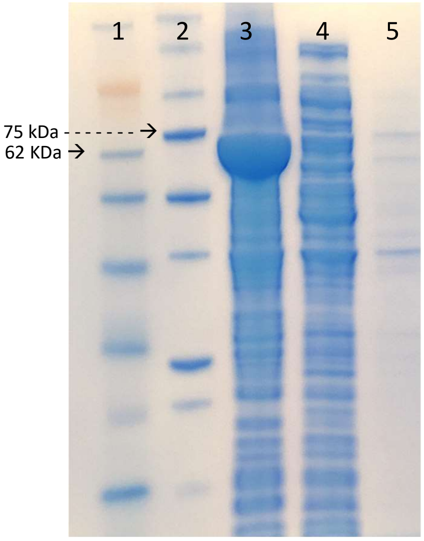

Figure S5. Illustrating the different timepoints (days) of the immunization trial, related to STAR Methods.

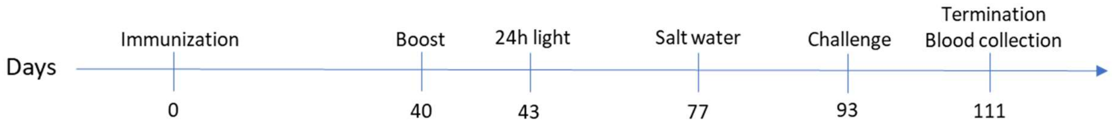

Table S1. Phylogenetic analysis of LsPxtl-1 blasted in the Uniprot database [1], related to Figure 2, Figure S3A. All species belongs to phylum Arthropoda.

Id Uniprot	Species	Subphylum	Class	Protein	%Identity
A0A553NVS2	Tigriopus californicus	Crustaceans	Hexanauplia	Uncharacterized protein	72.2%
A0A164VLV1	Daphnia magna	Crustaceans	Branchiopoda	Uncharacterized protein	49.2%
E9FWK3	Daphnia pulex	Crustaceans	Branchiopoda	Uncharacterized protein	48.4%
A0A1D2NGG8	Orchesella cincta	Hexapoda	Collembola	Peroxidasin	46.6%
A0A553N894	Tigriopus californicus	Crustaceans	Hexanauplia	Uncharacterized protein	45.7%
E0VJ44	Pediculus humanus subsp. cor.	Hexapoda	Insecta	Uncharacterized protein	45.3%
A0A6L2PEN3	Coptotermes formosanus	Hexapoda	Insecta	Uncharacterized protein	45.9%
E9FUG3	Daphnia pulex	Crustaceans	Branchiopoda	Uncharacterized protein	45.1%
T1KSA2	Tetranychus urticae	Chelicerata	Arachnida	Uncharacterized protein	43.5%
A0A087U0U3	Stegodyphus mimosarum	Chelicerata	Arachnida	Peroxidasin-like protein	44.3%
T1IHP0	Strigamia maritima	Myriapoda	Chilopoda	Uncharacterized protein	41.7%
T1HWA1	Rhodnius prolixus	Hexapoda	Insecta	Peroxidasin	44.1%
A0A6P8Z1S4	Thrips palmi	Hexapoda	Insecta	Uncharacterized protein LOC117646626	44.8%
A0A6J1S5G9	Frankliniella occidentalis	Hexapoda	Insecta	Peroxidasin homolog	45.5%
A0A7E4RH46	Cimex lectularius	Hexapoda	Insecta	Uncharacterized protein	44.2%
D6WXZ9	Tribolium castaneum	Hexapoda	Insecta	Uncharacterized protein	45.1%
A0A7J6ZGI4	Lamprigera yunnana	Hexapoda	Insecta	Uncharacterized protein	44.9%
A0A443S9N9	Leptotrombidium deliense	Chelicerata	Arachnida	Chorion peroxidase-like protein	42.5%
A0A1W4XF82	Agrilus planipennis	Hexapoda	Insecta	Uncharacterized protein LOC108741220	44.8%
A0A2J7RIR8	Cryptotermes secundus	Hexapoda	Insecta	Uncharacterized protein	47.0%
A0A6A4JD52	Apolygus lucorum	Hexapoda	Insecta	Uncharacterized protein	44.1%
A0A6P7FKB1	Diabrotica virgifera virgifera	Hexapoda	Insecta	Chorion peroxidase	44.7%
A0A482VGY8	Asbolus verrucosus	Hexapoda	Insecta	Uncharacterized protein	45.7%
A0A7J6ZSM9	Abscondita terminalis	Hexapoda	Insecta	Uncharacterized protein	43.9%
A0A5N4ALX8	Photinus pyralis	Hexapoda	Insecta	Uncharacterized protein	44.3%

Table S2. Phylogenetic analysis of LsPxtl-1 blasted in the Redoxibase database[3], related to Figure 2 A.

Id Redoxibase	Species	Phylum (Subphylum)	Class	Protein	Identity %	Alnlength
4144	Caenorhabditis elegans	Nematoda	Chromadorea	Invertebrate peroxinectin	35	1043
4279	Caenorhabditis briggsae	Nematoda	Chromadorea	Invertebrate peroxinectin	36	1018
4143	Caenorhabditis elegans	Nematoda	Chromadorea	Invertebrate peroxinectin	35	1051
4278	Caenorhabditis briggsae	Nematoda	Chromadorea	Invertebrate peroxinectin	34	1015
4281	Caenorhabditis briggsae	Nematoda	Chromadorea	Invertebrate peroxinectin	31	1020
4145	Caenorhabditis elegans	Nematoda	Chromadorea	Invertebrate peroxinectin	31	1021
4126	Fenneropenaeus chinensis	Arthropoda (Crustacea)	Malacostraca	Invertebrate peroxinectin	36	437
3672	Penaeus monodon	Arthropoda (Crustacea)	Malacostraca	Invertebrate peroxinectin	36	437
3670	Apis mellifera	Arthropoda (Hexapoda)	Insecta	Invertebrate peroxinectin	37	474
5841	Nasonia vitripennis	Arthropoda (Hexapoda)	Insecta	Invertebrate peroxinectin	35	499
4290	Tribolium castaneum	Arthropoda (Hexapoda)	Insecta	Invertebrate peroxinectin	35	493
4105	Pacifastacus leniusculus	Arthropoda (Crustacea)	Malacostraca	Invertebrate peroxinectin	32	504
5842	Nasonia vitripennis	Arthropoda (Hexapoda)	Insecta	Invertebrate peroxinectin	36	471
4127	Lytechinus variegatus	Echinodermata	Echinoidea	Invertebrate peroxinectin	35	470
3371	Lytechinus variegatus	Echinodermata	Echinoidea	Peroxidasin	34	504
4128	Hemicentrotus pulcherrimus	Echinodermata	Echinoidea	Invertebrate peroxinectin	35	470
7615	Saccoglossus kowalevskii	Hemichordata	Enteropneusta	Peroxidasin	33	520
10036	Acromyrmex echinatior	Arthropoda (Hexapoda)	Insecta	Peroxidasin	34	509
7622	Saccoglossus kowalevskii	Hemichordata	Enteropneusta	Peroxidasin	33	507
4288	Caenorhabditis briggsae	Nematoda	Chromadorea	Peroxidasin	31	588
4101	Strongylocentrotus purpuratus	Echinodermata	Echinoidea	Peroxidasin	33	509
4142	Caenorhabditis elegans	Nematoda	Chromadorea	Peroxidasin	33	507
3553	Drosophila melanogaster	Arthropoda	Insecta	Invertebrate peroxinectin	34	499
4148	Euprymna scolopes	Mollusca	Cephalopoda	Invertebrate peroxinectin	34	482
7648	Danio rerio	Chordata	Actinopterygii	Peroxidasin	40	430

Table S3. Phylogenetic analysis of LsPxtl-2 blasted in the Uniprot database [1], related to Figure 2, Figure S3B.

Id Uniprot	Species	Phylum (Subphylum)	Class	Protein	Identity
A0A553PB46	Tigriopus californicus	Arthropoda (Crustacea)	Hexanauplia	Uncharacterized protein	42.0%
A0A553PDD1	Tigriopus californicus	Arthropoda (Crustacea)	Hexanauplia	Uncharacterized protein	40.1%
A0A553PS73	Tigriopus californicus	Arthropoda (Crustacea)	Hexanauplia	Uncharacterized protein	38.1%
A0A553PR45	Tigriopus californicus	Arthropoda (Crustacea)	Hexanauplia	Uncharacterized protein	40.2%
A0A553PPT1	Tigriopus californicus	Arthropoda (Crustacea)	Hexanauplia	Uncharacterized protein	35.5%
A0A553PPR4	Tigriopus californicus	Arthropoda (Crustacea)	Hexanauplia	Uncharacterized protein	37.4%
A0A553PPJ4	Tigriopus californicus	Arthropoda (Crustacea)	Hexanauplia	Uncharacterized protein	35.3%
A0A553P656	Tigriopus californicus	Arthropoda (Crustacea)	Hexanauplia	Uncharacterized protein	35.4%
A0A226EHM9	Folsomia candida	Arthropoda (Hexapoda)	Collembola	Peroxidasin	39.4%
A0A226DH01	Folsomia candida	Arthropoda (Hexapoda)	Collembola	Chorion peroxidase	37.1%
A0A6J1T3K6	Frankliniella occidentalis	Arthropoda	Insecta	Peroxidase-like	35.4%
A0A6P4XZE9	Branchiostoma belcheri	Chordata	Leptocardii	thyroid peroxidase-like	37.5%
A0A1D2MN13	Orchesella cincta	Arthropoda	Collembola	Peroxidasin	37.1%
A0A1D2MWC0	Orchesella cincta	Arthropoda	Collembola	Chorion peroxidase	37.8%
A0A1D2MGG6	Orchesella cincta	Arthropoda	Collembola	Chorion peroxidase	34.4%
T1J5V8	Strigamia maritima	Arthropoda (Myriapoda)	Chilopoda	Uncharacterized protein	36.4%
E0VGJ5	Pediculus humanus subsp. cor…	Arthropoda (Hexapoda)	Insecta	Chorion peroxidase precursor, putative	38.1%
A0A226EH62	Folsomia candida	Arthropoda (Hexapoda)	Collembola	Chorion peroxidase	36.5%
A0A6P8YXE0	Thrips palmi (Melon thrips)	Arthropoda	Insecta	Peroxidase-like	35.0%
T1J5V9	Strigamia maritima	Arthropoda (Myriapoda)	Chilopoda	Uncharacterized protein	37.1%
A0A6P4ZNM3	Branchiostoma belcheri	Chordata	Leptocardii	Peroxidasin homolog	38.8%
A0A6G0TDN6	Aphis glycines	Arthropoda	Insecta	Uncharacterized protein	34.6%
A0A0P5DZW0	Daphnia magna	Arthropoda	Branchiopoda	Chorion peroxidase	37.8%
A0A1W0XF69	Hypsibius dujardini	Tardigrada	Eutardigrada	Chorion peroxidase	37.2%
J9JMG8	Acyrthosiphon pisum	Arthropoda (Hexapoda)	Insecta	Clip domain-containing protein	34.4%

Table S4. Phylogenetic analysis of LsPxtl-2 blasted in the Redoxibase database [3], related to Figure 2B.

ID Redoxibase	Latin name	Phylum (Subphylum)	Class	Protein	% Identity	Alnlength
5841	Nasonia vitripennis	Arthropoda (Hexapoda)	Insecta	Invertebrate peroxinectin	34	597
4126	Fenneropenaeus chinensis	Arthropoda (Crustacea)	Malacostraca	Invertebrate peroxinectin	34	590
5816	Monodelphis domestica	Chordata (Craniata)	Mammalia	Eosinophil peroxidase	36	595
3672	Penaeus monodon	Arthropoda (Crustacea)	Malacostraca	Invertebrate peroxinectin	34	590
4105	Pacifastacus leniusculus	Arthropoda (Crustacea)	Malacostraca	Invertebrate peroxinectin	34	579
3366	Rattus norvegicus	Chordata (Craniata)	Mammalia	Eosinophil peroxidase	36	591
5805	Equus caballus	Chordata (Craniata)	Mammalia	Eosinophil peroxidase	35	593
6440	Tupaia belangeri	Chordata (Craniata)	Mammalia	Eosinophil peroxidase	36	593
3346	Mus musculus	Chordata (Craniata)	Mammalia	Eosinophil peroxidase	35	593
7971	Ailuropoda melanoleuca	Chordata (Craniata)	Mammalia	Eosinophil peroxidase	36	587
4279	Caenorhabditis briggsae	Nematoda	Chromadorea	Invertebrate peroxinectin	34	603
4044	Macaca mulatta	Chordata (Craniata)	Mammalia	Eosinophil peroxidase	36	594
3317	Homo sapiens	Chordata (Craniata)	Mammalia	Eosinophil peroxidase	35	595
7792	Callithrix jacchus	Chordata (Craniata)	Mammalia	Eosinophil peroxidase	34	612
7796	Microcebus murinus	Chordata (Craniata)	Mammalia	Eosinophil peroxidase	36	596
4040	Gallus gallus	Chordata (Craniata)	Aves	Non mammalian vertebrate peroxidase	36	591
7789	Otolemur garnettii	Chordata (Craniata)	Mammalia	Eosinophil peroxidase	36	594
3367	Pan troglodytes	Chordata (Craniata)	Mammalia	Eosinophil peroxidase	35	595
7684	Gorilla gorilla	Chordata (Craniata)	Mammalia	Eosinophil peroxidase	35	595
4144	Caenorhabditis elegans	Nematoda	Chromadorea	Invertebrate peroxinectin	33	603
7972	Danio rerio	Chordata (Craniata)	Actinopterygii (Superclass)	Non mammalian vertebrate peroxidase	34	594
4128	Hemicentrotus pulcherrimus	Echinodermata	Echinoidea	Invertebrate peroxinectin	33	557
4045	Mesocricetus auratus	Chordata (Craniata)	Mammalia	Lactoperoxidase	35	597
3371	Lytechinus variegatus	Echinodermata	Echinoidea	Peroxidasin	36	579
3354	Bos taurus	Chordata (Craniata)	Mammalia	Myeloperoxidase	36	587

Table S5. Primer used for RACE, PCR amplification, TA cloning, qPCR, and restriction digestion for DNA and protein production in vector, pcDNA and pET32c respectively, related to STAR Methods. F: forward, R: reverse, fr: fragment, Tm: annealing temperature.

	Forward	Reverse	Forward sequence	Reverse sequence	Tm (C°)
RACE	LsPxtl-1 F	LsPxtl-1 R	Ggatcactgtctcctctcctctcgat	Ggtcgtagtggtggaattggcac	
RACE	LsPxtl-2 F	LsPxtl-2 R	Cgtcgaaggaacatccacatcagtatgcac	Gtcgcctctgttttcgttgctgctgcat	67/69
TA cloning	LsPxtl-1 F	LsPxtl-1 R	Ggtaagagcctcccatctcc	Tgaataaatccacatcatccaca	58
TA cloning	LsPxtl-2 F	LsPxtl-2 R	Gtggatgttccttcgacgat	Ggcatgaaccaaaaatgctt	58
RNAi Without T7	LsPxtl-1 F	LsPxtl-1 R	Acctggtcagcaacgacttggc	Ctggttctccataggcgtagca	
RNAi With T7	LsPxtl-1 F	LsPxtl-1 R	Taatacgactcactatagggagaacctggtcagcaacgacttggc	Taatacgactcactatagggagaccaatgatccgaggaaggaact	
RNAi Without T7	LsPxtl-2 F	LsPxtl-2 R	Tcaagcgacgaggaattattgc	Caagatctgctccatacggctt	
RNAi With T7	LsPxtl-2 fr 1 F	LsPxtl-2 fr R	Taatacgactcactatagggagatcaagcgacgaggaattattgc	Taatacgactcactatagggagatttttcccctaataacgtcgga	
RNAi With T7	LsPxtl-2 fr 2 F	LsPxtl-2 fr 2 R	Taatacgactcactatagggagacaagatctgctccatacggctt	Taatacgactcactatagggagagcatgaatgagtctggcttaaactt	
qPCR/RNAi	LsPxtl-1 F	LsPxtl-1 R	Gctgatgtttcccatctacatggacg	gcgcagagtcacaaccacgaca	
qPCR/RNAi	LsPxtl-2 F	LsPxtl-2 R	Tgcatactgatgtggatgttccttcg	gcctcgtgggaattgggaacaa	
qPCR/ Ontogeny	LsPxtl-1 F	LsPxtl-1 R	Gctgatgtttcccatctacatggacg	gcgcagagtcacaaccacgaca	
qPCR/ Ontogeny	LsPxtl-2 F	LsPxtl-2 R	Tgcatactgatgtggatgttccttcg	gcctcgtgggaattgggaacaa	
pcDNA	BamH I-LsPxtl-1 F<br>BaM Peron LsPxtl-1 F	Hind III- LsPxtl-1 R<br>Hin peron LsPxtl-1 R2	Acgcggatccatgctgatgtttccca	acgcaagctttacttgtttcagtctt	68
pcDNA	BamH I- LsPxtl-2 F<br>BaM Peron LsPxtl-2 F	Hind III- LsPxtl-2 R Hin peron LsPxtl-2 R2	Acgcggatccatggtaagccaatggg	acgcaagcttgaataaattaagattc	68
pET32c	BamH I- LsPxtl-1 F<br>BaM Peron LsPxtl-1 F3	Hind III- LsPxtl-1 R<br>Hin peron LsPxtl-1 R3	Acgcggatccgaatgctgatgtttccca	acgcaagcttagtacttgtttcagtctt	68
pET32c	BamH I- LsPxtl-2 F BaM Peron LsPxtl-2 F3	Hind III- LsPxtl-2 R Hin peron LsPxtl-2 R3	Acgcggatccgaatggtaagccaatggg	acgcaagcttaggaataaattaagattc	68

Table S6. Custom LNA mRNA detection probes with double DIG labelling from Qiagen used for *in situ* hybridization, related to STAR Methods.

Probe name	Sequence 5'- 3'
LsPxtl-1	/5DiGN/ATGAGCATAAGAGTGTATCGT/3DiG_N/
LsPxtl-2	/5DiGN/AGACGCAATCGTCGAAGGAACA/3DiG_N/
Positive (Beta-actin)	/5DiGN/CTCATTGTAGAAGGTGTGGTGCCA/3DiG_N/
Negative (Scramble-ish)	/5DiGN/GTGTAACACGTCTATACGCCCA/3DiG_N/

Table S7. Overview of vaccine groups, type of vaccine, boosting, injection route, dosage given to the salmon and number of fish sampled in the experiment, related to STAR Methods.

Groups	Type	Boost	Administrations route	Dose	No of fish
LsPxtl-1	DNA	-	IM	20 µg/fish	42
LsPxtl-2	DNA	-	IM	20 µg/fish	37
LsPxtl-1+2	DNA, protein boost (LsPxtl-2)	50 % of group	IM (IP boost)	20 µg/fish (10 µg/fish each variant),<br>50 µg/fish boost	8<br>10 (boost)
PBS	PBS	-	IP		35

Supplemental references:

1. Consortium TU: UniProt (2020). the universal protein knowledgebase in 2021. Nucleic Acids Res. *49*(D1):D480-D489.
2. Lemoine, F., Correia, D., Lefort, V., Doppelt-Azeroual, O., Mareuil ., Cohen-Boulakia, S., Gascuel, O. (2019). NGPhylogeny.fr: new generation phylogenetic services for non-specialists. Nucleic Acids Res. *47*(W1):W260-W265.
3. Savelli, B., Li, Q., Webber, M., Jemmat, A.M., Robitaille, A., Zamocky, M., Mathé, C., Dunand, C. (2019). RedoxiBase: A database for ROS homeostasis regulated proteins. Redox Biol. *26*:101247.
